# Capillary Gas Chromatographic Separation Performances of a Tetraphenyl Porphyrin Stationary Phase

**DOI:** 10.3389/fchem.2022.800922

**Published:** 2022-02-23

**Authors:** Yuan Yan, Zhenzhong Wang, Zitong Zhang, Zhen He, Lun Luo, Jing Fan

**Affiliations:** School of Pharmaceutical Sciences, Hubei Key Laboratory of Wudang Local Chinese Medicine Research, Hubei Provincial Technology and Research Center for Comprehensive Development of Medicinal Herbs, Hubei University of Medicine, Shiyan, China

**Keywords:** gas chromatography, stationary phase, tetraphenyl porphyrin, selectivity, separation mechanism

## Abstract

Tetraphenyl porphyrin (TPP) has enormous potential for use as gas chromatography stationary phases because it has a distinctive extended π–π conjugated coplanar structure and a range of interesting properties such as a good solubility in dichloromethane, high melting point, and good thermal stability. In this work, a TPP column was successfully prepared using a static method. The column was nonpolar and had a high efficiency. The chromatographic selectivity of the TPP column was assessed. The TPP column showed superiority retention and higher resolution for alicyclic, aromatic molecules through ring matching and π-π stacking interaction comparable to HP-5MS column. The unique mechanisms through which the TPP column retained polychlorinated biphenyls allowed the peak pair of 2,2ʹ,5-trichlorobiphenyl and 4,4ʹ-dichlorobiphenyl to be resolved better on the TPP column than the HP-5MS column. The TPP column was thermally stable even at 260°C for 2 h and gave results of a high degree of precision (run-to-run and column-to-column) with relative standard deviations <0.05% and <4.96%, respectively. The results indicated that porphyrin derivatives will be useful gas chromatography stationary phases.

## Introduction

**FIGURE 1:**
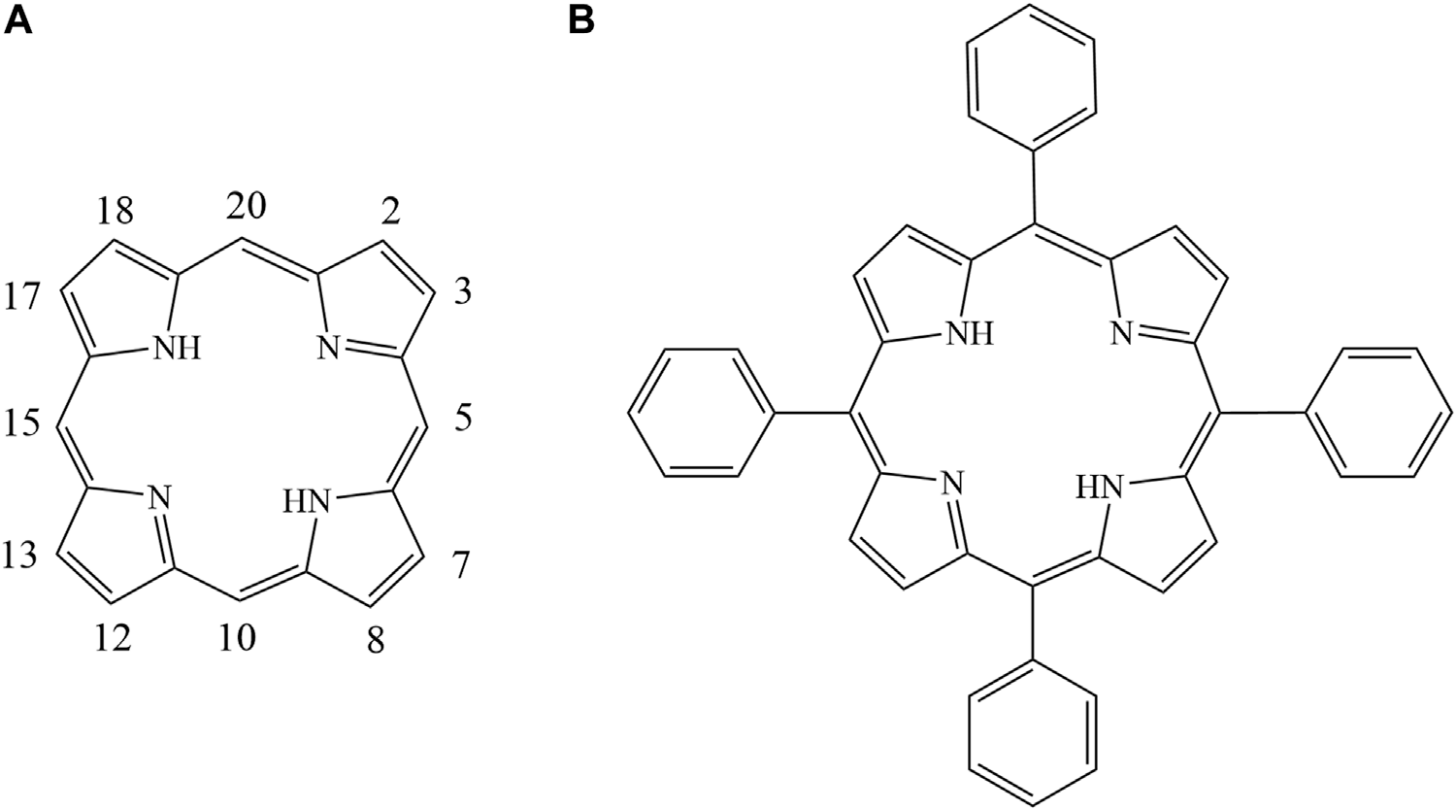
Structures of tetraphenyl porphyrin **(A)**. 5,10, 15, 20 meso-positions; **(B)**. 2,3,7,8,12, 13, 17,18 β-positions).

Porphyrins are multifunctional aromatic macrocycles composed of homologs and derivatives of porphine with groups substituted on the outside ring. As shown in Figure 1, porphine has a large electron-rich aromatic ring with an 18-electron π-conjugated system (Rovira et al., 2001; Koizumi et al., 2004). Various porphyrins can be synthesized by adding substituents with different structures and by adding different numbers of substituents at the meso- and β-positions of porphine (Figure 1) (Rothemund, 1935; Rothemund, 1936; Rothemund, 1939; Gottfried, 2015). The structures and properties of porphyrins make them important in various fields including medicine (Králová et al., 2010; Ethirajan et al., 2011), catalysis (Rezaeifard and Jafarpour, 2014; Zhao et al., 2019), the environment and energy(Goswami et al., 2018), and analytical chemistry (Biesaga et al., 2000; Paolesse et al., 2017; Masih et al., 2018). Porphyrins have been used to prepare materials for separating and enriching metal ions, peptides, phytosterols, and proteins in complex biological matrices(Hu et al., 2002; Jiang et al., 2017; Wang et al., 2017; Wei et al., 2017; Zhang et al., 2017; Zhang et al., 2018; Peng et al., 2019). Previously research employed Porphyrins combing with silica as stationary phases for liquid chromatographic separation of aromatic sulfonates, fullerenes, and polycyclic aromatic hydrocarbons (PAHs), but have given poor resolution and serious peak tailing (Kibbey and Meyerhoff, 1993; Xiao and Meyerhoff, 1996; Coutant et al., 1998; Biesaga et al., 1999; Chen et al., 2001).

Tetraphenyl porphyrin (TPP) (Figure 1) was first synthesized and purified more than a century ago via condensation of pyrrole and benzaldehyde using a method described by Adler and Lindsey (Adler et al., 1964; Adler et al., 1967). As shown in Figure 1, TPP has four phenyl moieties in meso positions with an extended π–π conjugated coplanar structure, which have good solubility in dichloromethane, a high melting point and outstanding thermal stability. TPP may therefore be a useful new stationary phase for use in capillary gas chromatography (GC) columns. To the best of our knowledge, TPP has not previously been used as a GC stationary phase. We have previously used calix[4]pyrroles, which have similar structures to porphyrins, as GC stationary phases (Fan et al., 2014). However, TPP has sp^2^ hybridized carbons in the meso positions but calix [4] pyrroles have sp^3^ hybridized carbon atoms in the meso positions. TPP has large-plane π–π conjugated ring structures that are aromatic, but calix [4] pyrroles do not. TPP may therefore give separation characteristics different from currently available GC stationary phases.

In the study described here, TPP was used for the first time as a capillary GC stationary phase. A static method was successfully used to coat TPP onto a capillary column pretreated with sodium chloride (NaCl). The separation performance of the TPP column was investigated using specially screened mixtures including a Grob mixture; a mixture of six esters and *n*-alkanes with similar boiling points; a mixture of cycloalcohols and straight-chain alcohols; a mixture of cycloaldehydes and straight-chain alcohols; a mixture of polychlorinated biphenyls (PCBs), polycyclic aromatic hydrocarbons (PAHs), phthalate esters (PAEs), and *n*-alkanes; and a mixture of 15 analytes. The TPP stationary phase separation mechanism was investigated by assessing separation parameters including the resolution and retention factors of pairs of analytes such as diethyl phthalate and fluorine, 2,2ʹ,5-trichlorobiphenyl and 4,4ʹ-dichlorobiphenyl at different temperatures. Other chromatographic parameters including the column efficiency, polarity, thermal stability, and repeatability of separation were also assessed. The same tests were performed using a commercial HP-5MS column with a similar polarity to the TPP column for comparative purposes.

## Materials and Methods

### Materials and Apparatus

All the reagents used were of analytical grade or better. Tetraphenyl porphyrin was purchased from Alfa Aesar (Heysham, United Kingdom). Benzene, 1-butanol, 1-nitropropane, 2-pentanone, and pyridine were purchased from Alfa Chemical Co. (Tianjin, China). 2,6-Dichlorobiphenyl, 4,4ʹ-dichlorobiphenyl, diethyl phthalate, 2,2ʹ,5-trichlorobiphenyl, and 2,3,4-trichlorobiphenyl were purchased from Sigma-Aldrich (St. Louis, MO, Uniterd States). Dibutyl phthalate, dipentyl phthalate, fluoranthene, fluorene, and pyrene were purchased from Aladdin Chemistry Co. (Shanghai, China). The other reagents were purchased from Beijing Chemical Reagent Company (Beijing, China). Each analyte was dissolved in dichloromethane at a concentration of 5 μg ml^−1^ for the separations. Untreated fused-silica capillary tubing (0.25 mm i.d.) was purchased from Yongnian Ruifeng Chromatogram Apparatus Co. (Hebei, China). An HP-5MS capillary column (10 m long, 0.25 mm i.d.) was purchased from Agilent Technologies (Santa Clara, CA, Uniterd States).

The analyses were performed using a Trace 1300 gas chromatograph (Thermo Fisher Scientific, Waltham, MA, Uniterd States) with an RSH autosampler, a split/splitless injector, a flame ionization detector. The GC parameters were: carrier gas (high purity (99.999%) nitrogen) flow rate 1 ml min^−1^, injector temperature 300°C, flame ionization detector temperature 300°C, injection volume 0.2 μl, and split mode injection (split ratio 50:1). The temperature program used to acquire a chromatogram is shown in the relevant figure caption below.

### Preparation of the TPP Capillary Column

A bare fused-silica capillary (10 m long, 0.25 mm i.d.) was kept at 200°C and purged with nitrogen for 3 h. Then, a saturated solution of NaCl in methanol was perfused and flowed through it. The next step, the inner wall of capillary was coated with TPP using the static method(Bouche and Verzele, 1968; Grob and Grob, 1976). TPP in dichloromethane (0.25% w/v) was injected into the capillary, then the whole capillary was coiled and incubated in a water bath at 40°C. One end of the capillary was sealed with soap while the other end was connected to a negative suction device to slowly remove the dichloromethane. To improve distribution uniformity, the TPP-coated capillary was heated from 40 to 180°C at 1°C min^−1^ and then kept at 180°C for 8 h while nitrogen was passed through the capillary at a flow rate of 1 ml min^−1^.

## Results and Discussion

### Characterization of the TPP Column

**FIGURE 2:**
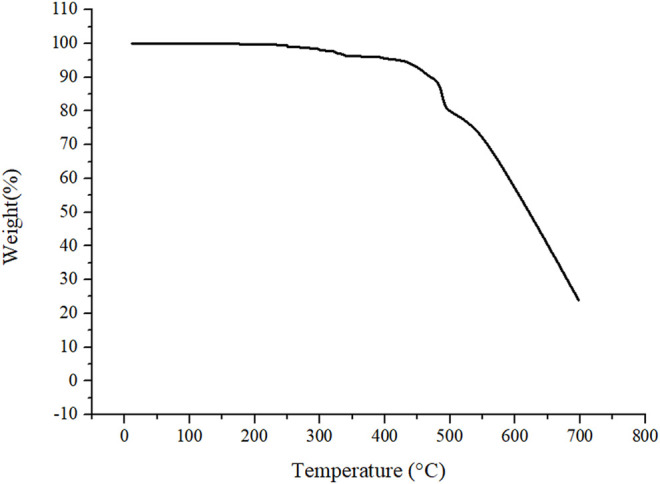
TGA curve of TPP from 20°C to 700°C at 10°C min^−1^ under nitrogen.

Thermal gravimetric analysis (TGA) was used to evaluate the thermal stability of TPP column. As shown in Figure 2, when the TPP column was heated to 309°C, only 2% weight was lost. It indicated that the thermal stability of the TPP column was good enough to be the GC stationary phase.

**FIGURE 3:**
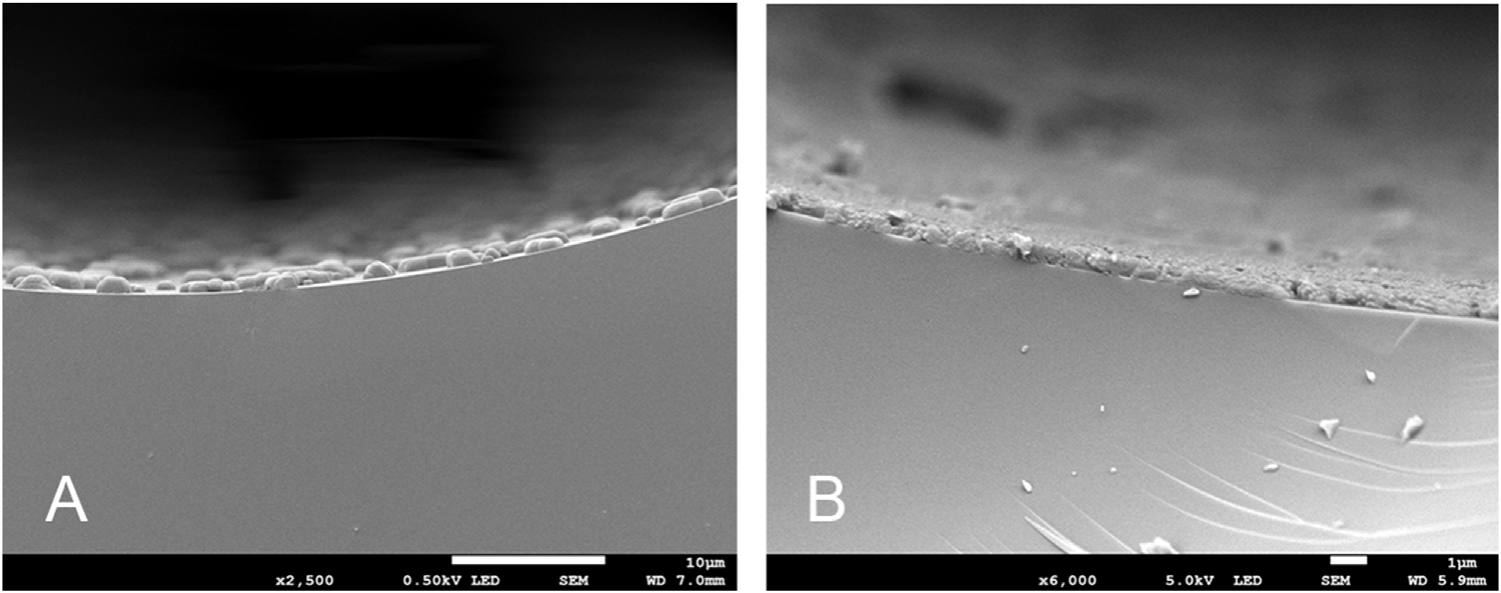
SEM image of the inner wall of the capillary column **(A)**. NaCl particle coated; **(B)**. TPP coated).

To verify uniformity of the entire length of the column, the cross-sections of several TPP capillary column segments at different positions were also characterized by scanning electron microscopy (SEM) as well as the cross-sections of the NaCl particle coated inner wall of the capillary column for comparison. Figure 3A clearly shows that inner wall of capillary column was successfully roughened by NaCl crystal particles uniformly. Meanwhile, the uniform and dense coatings were observed from the cross-section of the TPP column by SEM in Figure 3B and it indicated TPP deposited on the NaCl particle inner wall of capillary column homogeneously. The other SEM images of the different positions from the different segments also showed the coating similarly uniform and dense (see the Supplementary Material S1). The thickness of NaCl-TPP coating from the different segments were about 0.7–0.8 µm respectively attesting the thickness uniformity of the entire length of the column. Furthermore, the TPP layer thickness was about 0.16 µm calculated by the empirical formula, df=(dc/c)/400, where dc is the capillary inner diameter(mm) and c is the concentration of the stationary phase(%,w/v) (Shi et al., 2012; Shi et al., 2013).

The TPP column was evaluated to validate the separation capability in terms of column efficiency (Golay, 1957). The column efficiencies of the TPP and HP-5MS columns are shown in Table 1 which were determined using *n*-dodecane and naphthalene at 120°C and a flow rate of 1 ml min^−1^. The TPP column gave high efficiencies of 3,376 plates m^−1^ for *n*-dodecane and 2,717 plates m^−1^ for naphthalene, meaning that the TPP column was appropriate to perform subsequent separation tests.

**TABLE 1:** Column efficiencies (plates/m) of the TPP and commercial columns.

Column stationary phase	Column efficiency(n-dodecane)	Column efficiency (naphthalene)
TPP	3,376	2,717
HP-5MS	2,641	3,078

The TPP column was also evaluated to validate the separation capability in terms of column polarity. The McReynolds constant were determined from ΔI, defined as the differences between the retention indices of five probe compounds (benzene, *n*-butanol, 1-nitropropane, 2-pentanone, and pyridine) on the test stationary phase and squalane stationary phase at 120°C. The parameter $\bar{\Delta I}$ , the mean difference between the retention indices of the five probe compounds was more often to characterize the GC stationary phase polarity (McReynolds, 1970; Rajkó et al., 2005). When the value of $\bar{\Delta I}$ is less than 100, this stationary phase is considered non-polar. The McReynolds constants for the TPP column and commercial one HP-5MS are shown in Table 2. The $\bar{\Delta I}$ values of the TPP column and the HP-5MS column are 79.2 and 67 respectively, indicating that both are non-polar column and has similar polarity. The HP-5MS column was therefore used for subsequent tests to compare with the results of TPP column.

**TABLE 2:** McReynolds constants of the TPP and HP-5MS columns.

Stationary phase	X′	Y′	Z′	U′	S′	Sum	Average
I for TPP	769	672	705	715	756		
I for squalane	653	590	627	652	699		
△I for TPP	116	82	78	63	57	396	79.2
△I for HP-5MS	33	72	66	99	67	337	67

X´, benzene; Y´, n-butanol; Z´, 2-pentanone; U´, 1-nitropropane; S´, pyridine; Temperature, 120°C.

### Separation Performance of the TPP Stationary Phase

**FIGURE 4:**
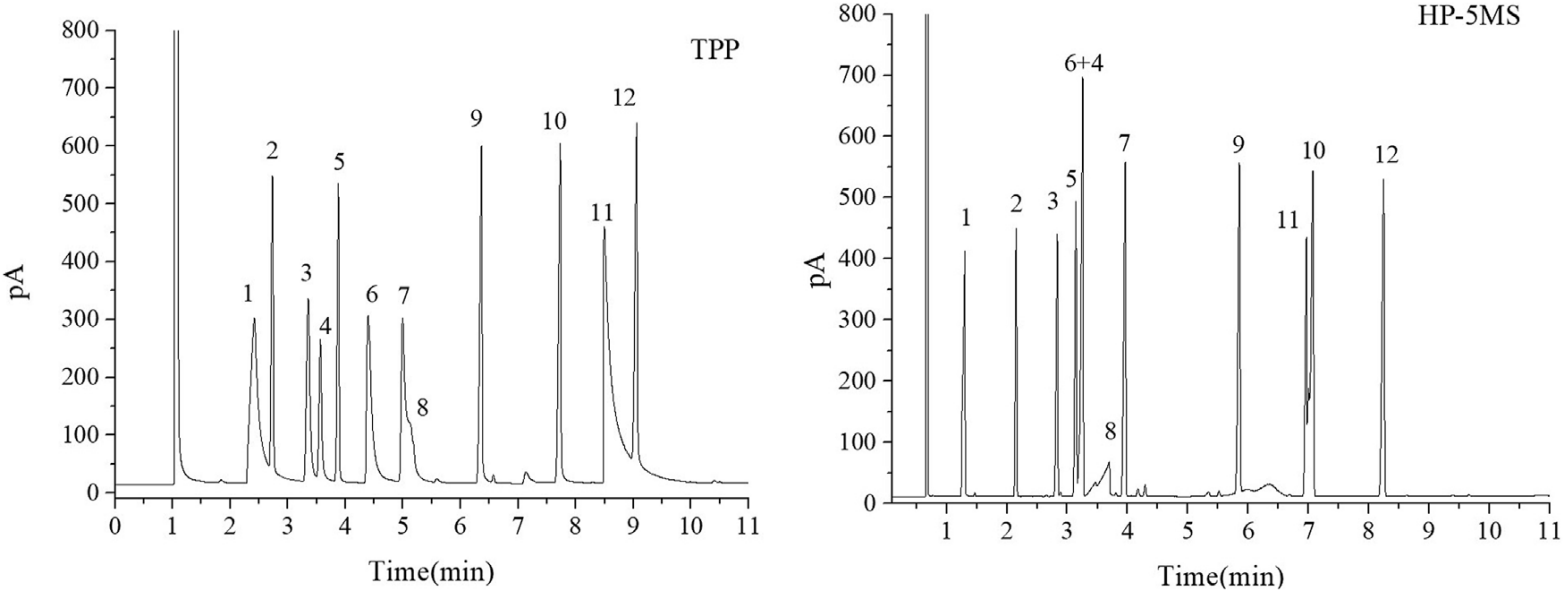
GC separation of the Grob mixture on the TPP and HP-5MS columns. Peaks:(1) 1,3-butanediol, (2) n-decane, (3) n-octanol, (4) n-nonanal, (5) n-undecane, (6) 2,6-dimethylphenol, (7) 2,6-dimethylaniline, (8) 2-ethylhexanoic acid, (9) methyl decanoate, (10) methyl undecanoate, (11) dicyclohexylamine, (12) methyl dodecanoate. Temperature program: 80°C (1 min) to 160°C at 10°C min^−1^.

**FIGURE 5:**
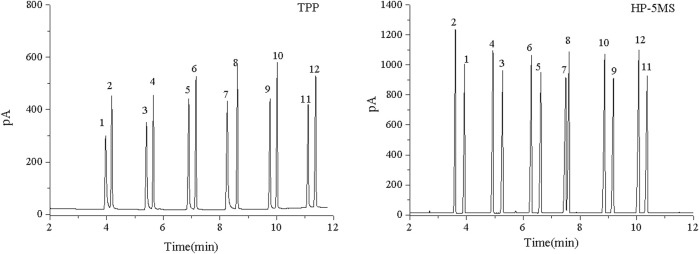
GC separation of the n-alkanes and esters mixture on the TPP and HP-5MS columns. Peak: (1) methyl heptanoate, (2) n-decane, (3) methyl octanoate, (4) n-undecane, (5) methyl nonoate, (6) n-dodecane, (7) p-methyl ethyl benzoate, (8) n-tridecane, (9) methyl undecanoate, (10) n-tetradecane, (11) methyl dodecanoate, (12) n-pentadecane. Temperature program: 60°C (1 min) to 160°C at 10°C min^−1^.

**FIGURE 6:**
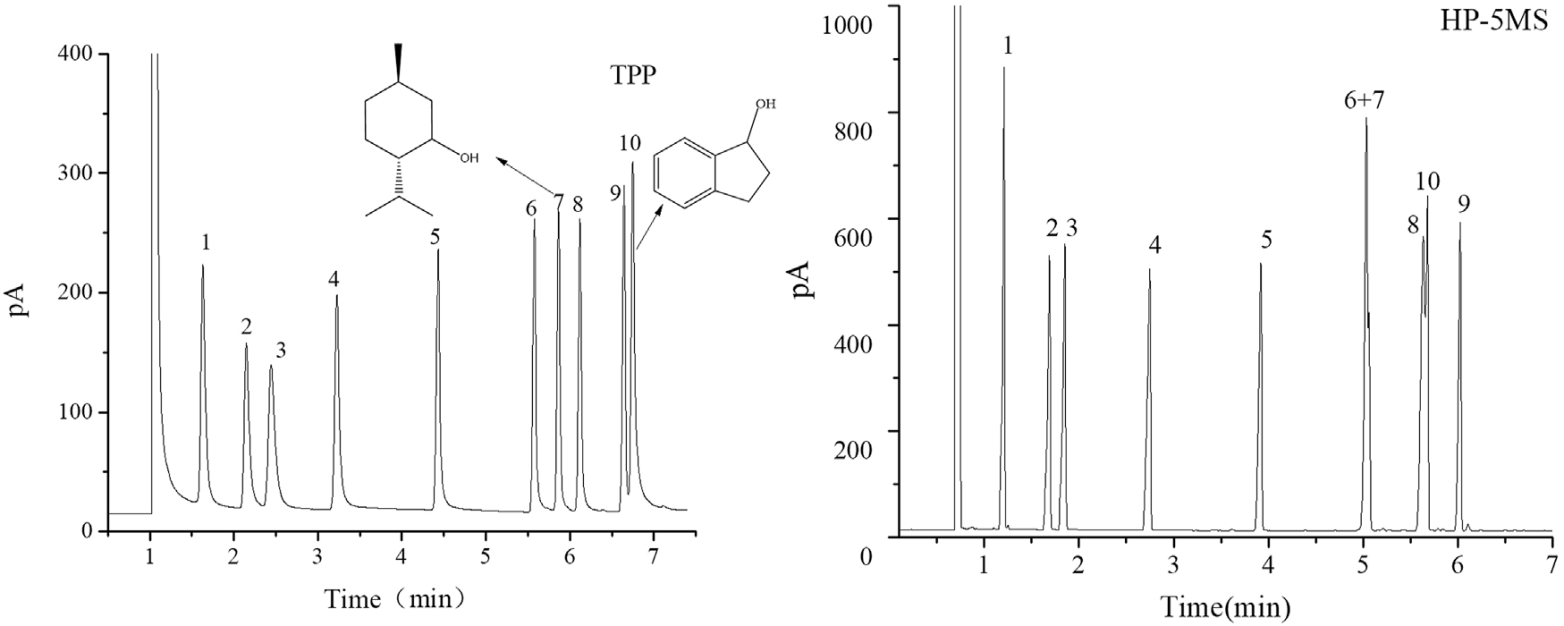
GC separation of the alcohols mixture on the TPP column and HP-5MS column. Peaks: (1) cyclopentanol, (2) n-hexanol, (3) cyclohexanol, (4) n-heptanol, (5) n-octanol, (6) n-nonanol, (7) D/L-menthol, (8) nerol, (9) n-decanol, (10) 1-indanol. Temperature program: 70°C (1 min) to 160 C at 15°C min^−1^.

**FIGURE 7:**
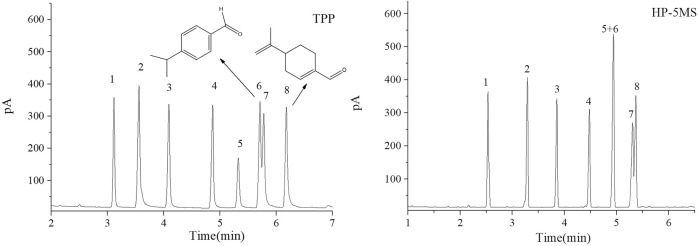
GC chromatograms for separations of a mixture of aldehydes on TPP and HP-5MS capillary columns. Peaks:(1) sinensal, (2) n-nonanal, (3) citronellal, (4) n-decanal, (5) geranial, (6) cuminaldehyde, (7) neral, (8) perillaldehyde. Temperature program: 80 °C (1 min) to 160 °C at 10 °C min−1.

**FIGURE 8:**
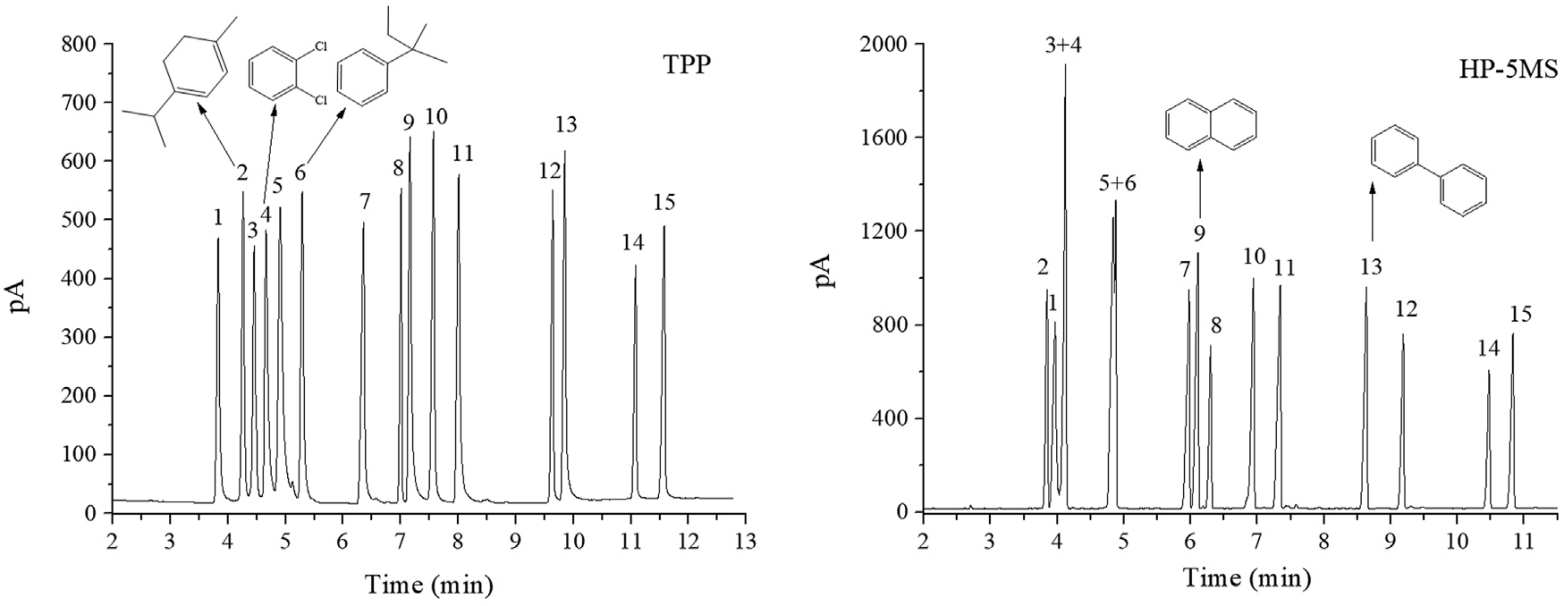
GC separation of a mixture of 15 analytes on TPP column and HP-5MS column. Peak: (1) methyl heptanoate, (2) α-terpinene, (3) ocimene, (4) o-dichlorobenzene, (5) fenchone, (6) tert-amylbenzene, (7) nonanol, (8) dodecane, (9) naphthalene, (10) neophyl chloride, (11) perillaldehyde, (12) methyl undecanoate, (13) biphenyl, (14) *cis*-nerolidol, (15) *trans*-nerolidol. Temperature program: 60°C (1 min) to 160°C at 10°C min−1.

**FIGURE 9:**
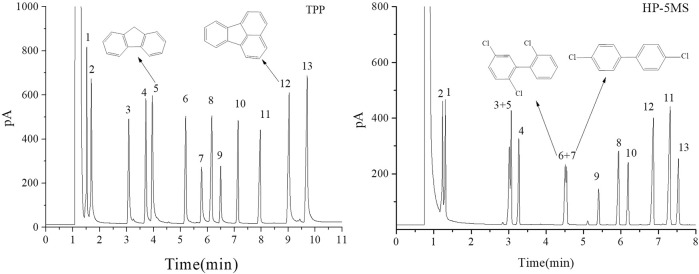
GC separation of the mixture of PCBs, PAHs, PAEs, and n-alkanes on the TPP column and HP-5MS column. Peak: (1) n-dodecane, (2) naphthalene, (3) diethyl phthalate, (4) 2,6-dichlorobiphenyl, (5) fluorene, (6) 2,2ʹ,5-trichlorobiphenyl, (7) 4,4ʹ-dichlorobiphenyl, (8) dibutyl phthalate, (9) 2,3,4-trichlorobiphenyl, (10) n-eicosane, (11) dipentyl phthalate, (12) fluoranthene, (13) pyrene. Temperature program: 160°C (1 min) to 260°C at 10°C min−1.

**FIGURE 10:**
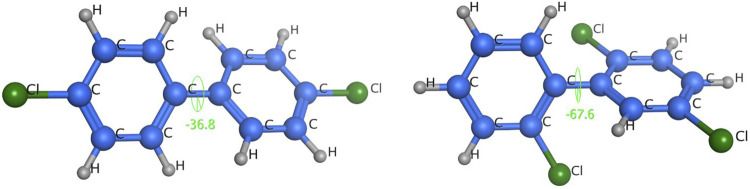
Geometric structures and dihedral angles of 2,2ʹ,5-trichlorobiphenyl and 4,4ʹ-dichlorobiphenyl.

**FIGURE 11:**
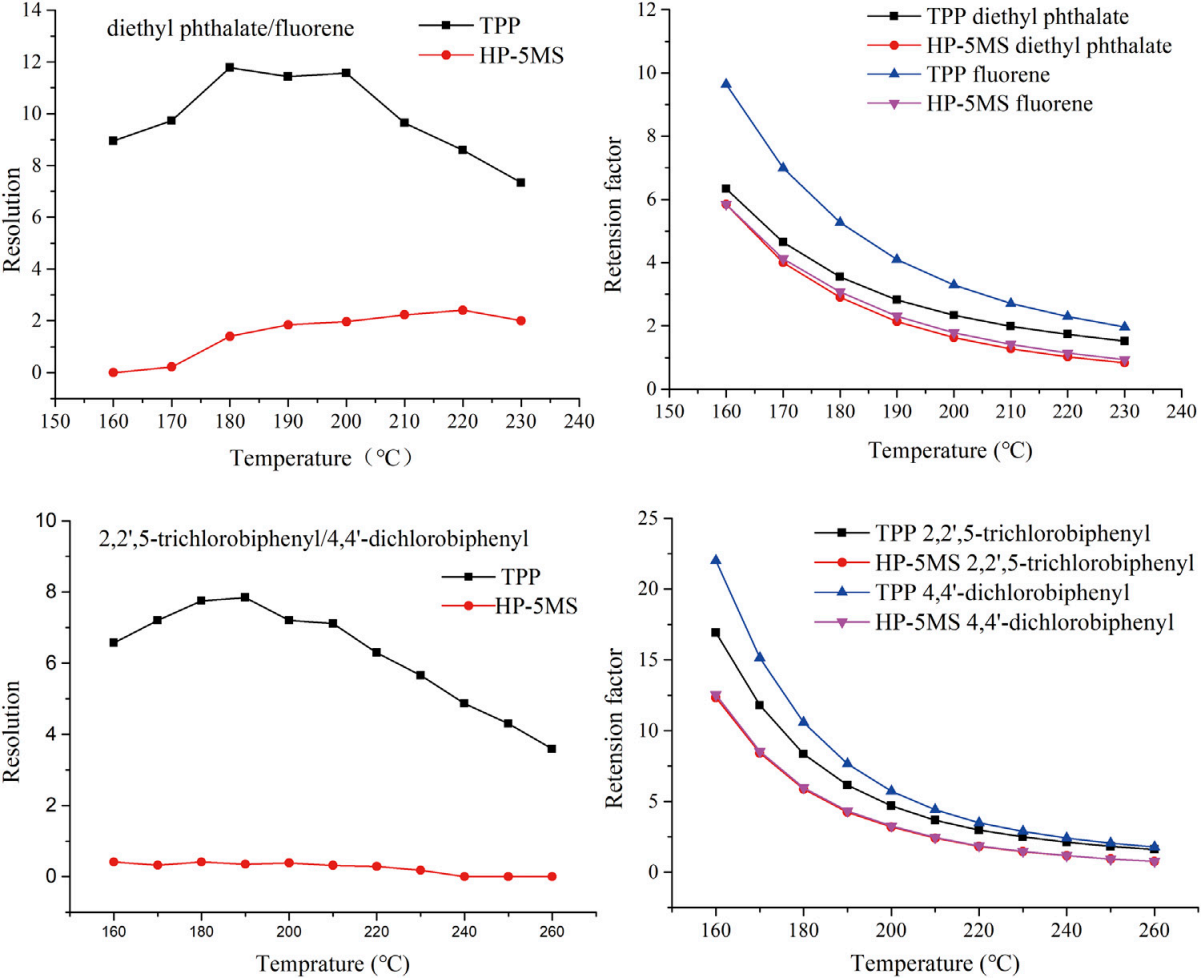
Effect of column temperature on resolution and retention factor of diethyl phthalate and fluorene (peaks 3 and 5) and of 2,2ʹ,5-trichlorobiphenyl and 4,4ʹ-dichlorobiphenyl (peaks 6 and 7) on TPP and commercial capillary columns.

**FIGURE 12:**
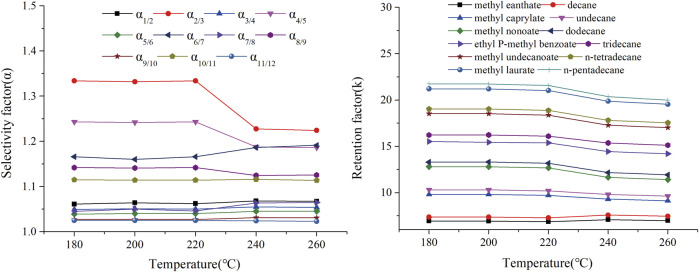
Effect of conditioning temperature on the retention factor (k) and selectivity factor (α) of n-alkane and ester mixtures on TPP column. Peaks: (1) methyl heptanoate, (2) decane, (3) methyl caprylate, (4) undecane, (5) methyl nonaoate, (6) dodecane, (7) p-methyl ethylbenzoate, (8) tridecane, (9) methyl undecanate, (10) n-tetradecane, (11) methyl laurate, (12) n-pentadecane.

The separation performance of the TPP column was assessed by analyzing various mixtures (a Grob mixture; a mixture of six esters and *n*-alkanes; mixtures of aldehydes and alcohols; a mixture of PCBs, PAHs, PAEs, and *n*-alkanes; and a mixture of 15 analytes). Chromatograms, separation parameters, and geometrical structures of relevant analytes are shown in Figures 4–12 and compounds structure were provided in Supplementary Material S2.

The Grob mixture contained 12 analytes and was used to comprehensively evaluate the chromatographic behavior of the GC column in terms of the peak shapes, chromatographic parameters, and elution order for the 12 analytes. The chromatograms for the Grob mixture acquired using the TPP and HP-5MS columns are shown in Figure 4. It can be seen that the TPP column gave baseline separation between most of the analytes and sharp symmetrical peaks for most of the analytes except 1,3-butanediol (peak 1), 2-ethylhexanoic acid (peak 8), and dicyclohexylamine (peak 11). These three compounds are strong proton donors, which would have caused slight peak broadening on the TPP column. The generally good separation indicated that there were hydrogen-bond and Lewis-acid adsorption sites on the inner walls of the TPP column and that the column had a good degree of inertness. The resolution was better for the *n*-undecane and 2,6-dimethylphenol (peaks 5 and 6) peak pair using the TPP column (R = 5.57) than the HP-5MS column (R = 1.84), probably because hydrogen-bonding interactions between 2,6-dimethylphenol and the N–H groups on the TPP stationary phase caused 2,6-dimethylphenol to be retained longer than *n*-undecane. The elution order of several peak pairs (e.g., 2,6-dimethylaniline and 2-ethylhexanoicacid (peaks 7 and 8) and methyl undecanoate and dicyclohexylamine (peaks 10 and 11) were opposite in the TPP column to that of the HP-5MS column. This may be due to the fact that the later eluting one in each pair had a stronger retention effect with TPP column, which was attributed to the hydrogen bonding between the analyte and the N-H group of the TPP. Notably, *n*-nonanal and *n*-undecane (peaks 4 and 5) were eluted in the opposite order from the TPP column and HP-5MS column but *n*-nonanal (peak 4) was well separated from the adjacent analytes by the TPP column but coeluted with 2,6-dimethylaniline (peak 6) using the HP-5MS column. The TPP column gave excellent separation and sharp peaks of monohydric alcohol (peak 3), *n*-alkanes (peak 5), fatty acid methyl esters (peaks 9, 10, and 12), monophenol (peak 6), and monoamine (peak 7), which supported the assertion that the TPP column is very efficient at separating strongly polar, medium polar, nonpolar, acidic, and basic compounds. In conclusion, the results above indicated that the TPP column was exceptionally inert and satisfactorily resolved the separation of the compounds of interest.

The mixture of six esters and *n*-alkanes was separated as well by the TPP column as by the HP-5MS column, and the chromatograms are shown in Figure 5. Interestingly, the *n*-alkanes and esters with similar boiling points eluted from the TPP column in the opposite order that they were eluted from the HP-5MS column. The TPP stationary phase had an extended π–π conjugated coplanar hydrophobic structure that interacted well with the hydrophobic *n*-alkanes, meaning that *n*-alkanes were more strongly retained by the TPP column than the HP-5MS column. Lone-pair-electron repulsion between the oxygen atoms in the esters and the nitrogen atoms in the TPP will have decreased the retention times of the esters on the TPP column. These results indicated that the TPP stationary phase had a particularly strong ability to retain *n*-alkanes and that *n*-nonanal and *n*-undecane (peaks 4 and 5 in Figure 4) in the Grob mixture were eluted in the opposite order from the TPP column and HP-5MS column.

The TPP column separated straight-chain alcohols in the Grob mixture well. These results and the extended π–π conjugated coplanar structure of the TPP stationary phase led us to select a mixture of alcohols including cycloalcohols and straight-chain alcohols to further study the TPP column performance. The results of the separation by TPP and HP-5MS columns are shown in Figure 6. Better resolutions and more symmetrical peak shapes were given for all of the alcohols by the TPP column than the HP-5MS column. In particular, the peak pairs *n*-nonanol and DL-menthol (peaks 6 and 7) and nerol and 1-indanol (peaks 8 and 10) were clearly separated by the TPP column but were eluted as single peaks from the HP-5MS column. The cycloalcohols DL-menthol (peak 7) and 1-indanol (peak 10) eluted later than the other alcohol in the peak pairs from the TPP column, and this led to the ability of the TPP column to resolve the peak pairs. DL-Menthol and 1-indanol may have eluted later than their peak pair compounds because of specific interactions between the TPP stationary phase and cyclic (aromatic and aliphatic ring) alcohols. 1-Indanol contains both an aromatic and an aliphatic ring and was more strongly retained by the TPP column than the HP-5MS column. In fact, n-decanol and 1-indanol (peaks 9 and 10) were eluted in the opposite order from the TPP column and HP-5MS column. The mixture of cycloaldehydes and straight-chain alcohols were separated well by the TPP column, which also indicated that the TPP stationary phase was selective for cyclic compounds. As shown in Figure 7, the geranial and cuminaldehyde (peaks 5 and 6) and neral and perillaldehyde (peaks 7 and 8) peak pairs were baseline separated by the TPP column but only partially resolved by the HP-5MS column. In summary, the extended π–π conjugated coplanar structure causes the TPP stationary phase to strongly and selectively retain cyclic molecules through ring matching or π–π stacking interactions between aromatic rings.

The results for the mixtures described above led us to investigate the retention of complex mixtures of 15 different analytes on the TPP stationary phase. Chromatograms of the analytes acquired using the TPP and HP-5MS columns are shown in Figure 8. The TPP column gave better resolutions and reversed elution sequences for some peak pairs compared with the HP-5MS column regardless of the sample complexity. In particular, the TPP column gave baseline resolution between the ocimene and *o*-dichlorobenzene (peaks 3 and 4; R = 0.85) and fenchone and *tert*-amylbenzene (peaks 5 and 6; R = 1.04) peak pairs. These peak pairs eluted as single peaks from the HP-5MS column because they have very similar boiling points (within 1–2°C). The elution sequences for some peak pairs, including methyl heptanoate and α-terpinene (peaks 1 and 2), dodecane and naphthalene (peaks 8 and 9), and methyl undecanate and biphenyl (peaks 12 and 13), were opposite on the TPP and HP-5MS columns. The good separation results described above were attributed to the particular ability of the TPP column to retain molecules containing aromatic rings. The TPP column also had more affinity than the HP-5MS column for the cyclic conjugated diolefine α-terpinene (peak 2), so heptanoate and α-terpinene (peaks 1 and 2) were resolved better by the TPP column than the HP-5MS column. This was attributed to π–π stacking interactions between the conjugated α-terpinene and TPP systems. The TPP column resolved *cis*- and *trans-*nerolidol (peaks 14 and 15) as well as did the HP-5MS column.

The results described above indicated that the extended π–π conjugated coplanar structure gave the TPP stationary phase a particular selectivity for monocyclic molecules. It was expected that TPP would be strongly selective for polycyclic π–π conjugated molecules such as PAHs and PCBs. A mixture of PCBs, PAHs, PAEs, and *n*-alkanes was therefore separated using the TPP and HP-5MS columns to investigate the selectivity of the TPP stationary phase further and to attempt to identify the mechanism(s) involved in separation by the TPP stationary phase. As shown in Figure 9, the TPP stationary phase was particularly selective for PAHs. This was indicated by the good resolution of diethyl phthalate from fluorene (peaks 3 and 5) given by the TPP column and the opposite elution sequences of various peak pairs including *n*-dodecane and naphthalene (peaks 1 and 2), 2,6-dichlorobiphenyl and fluorene (peaks 4 and 5), and diamyl phthalate and fluoranthene (peaks 11 and 12) given by the TPP and HP-5MS columns. The affinities between the PAHs and TPP would have been caused by the similar conjugate plane structures and large delocalized π-electron systems of TPP and PAHs causing intense π–π stacking interactions between TPP and PAHs. This would have cause PAHs to be more strongly retained by the TPP column than the HP-5MS column. Di-*n*-butyl phthalate and 2,3,4-trichlorobiphenyl (peaks 8 and 9) were also eluted in the opposite order from the TPP and HP-5MS columns. The chlorine substituents in a PCB molecule are strongly electron withdrawing and can cause the benzene ring in the PCB molecule to be electron deficient. PCBs can act as π-electron acceptors and cause strong π–π electron–donor–acceptor interactions with π-electron-rich TPP molecules, which act as π-electron donors (Luo et al., 2012). These π–π electron–donor–acceptor interactions would cause PCBs to be retained more strongly by the TPP column than the HP-5MS column, which would explain the di-*n*-butyl phthalate and 2,3,4-trichlorobiphenyl (peaks 8 and 9) elution sequence to be opposite for the TPP and HP-5MS columns. The PCBs 2,2ʹ,5-trichlorobiphenyl and 4,4ʹ-dichlorobiphenyl (peaks 6 and 7) were separated well by the TPP column but not by the HP-5MS column even though the boiling points are only 0.6 °C different. This indicated that the mechanism involved in the retention of PCBs by the TPP column may have been related to the planar structures of the PCBs.

As shown in Figure 10, 4,4ʹ-dichlorobiphenyl (peak 7 in Figure 9), which has a small dihedral angle (36.79°), is more planar than 2,2ʹ,5-trichlorobiphenyl, which has a larger dihedral angle (67.64°). This would mean that there would be more contact and therefore a stronger intermolecular force between 4,4ʹ-dichlorobiphenyl (peak 7 in Figure 9) and the TPP column than between 2,2ʹ,5-trichlorobiphenyl (peak 6 in Figure 9) and the TPP column. This would explain the good resolution between 2,2ʹ,5-trichlorobiphenyl and 4,4ʹ-dichlorobiphenyl (peaks 6 and 7 in Figure 9) achieved using the TPP column. The mechanism involved in the retention of PCBs by the TPP column was therefore related to PCB planarity caused by the PCB dihedral angle. Until now, 2,2ʹ,5-trichlorobiphenyl and 4,4ʹ-dichlorobiphenyl (peaks 6 and 7 in Figure 9) have not been successfully separated using any other GC stationary phase.

The column temperature strongly affects the ability of a GC column to separate analytes. The selectivity described above was investigated further by attempting to separate diethyl phthalate and fluorene (peaks 3 and 5 in Figure 9) and 2,2ʹ,5-trichlorobiphenyl and 4,4ʹ-dichlorobiphenyl (peaks 6 and 7 in Figure 9) at different column temperatures. As shown in Figure 11, the resolutions and retention factors were better for the TPP column than the HP-5 MS column at the temperatures that were tested. In particular, 2,2ʹ,5-trichlorobiphenyl and 4,4ʹ-dichlorobiphenyl (peaks 6 and 7 in Figure 9) were not baseline separated by the HP-5MS column at any of the test temperatures and the retention factors were basically the same. These results indicated the advantages of the TPP column over the HP-5MS column for separating persistent organic pollutants and indicated the differences between the separation mechanisms involved in chromatography using the TPP stationary phase and the HP-5MS polysiloxane stationary phase caused by the extended π–π conjugated coplanar structure of TPP.

### Thermal Stability and Repeatability

Thermal stability is essential to the practical use of a GC capillary column. A thermally stable capillary column is required for efficiently separating high-boiling-point compounds and for minimizing column bleed. The thermal stability of the TPP column was therefore investigated by assessing variations in the separation parameters of various analytes after the column had been aged at 180, 200, 220, 240, and 260°C for 2 h. The effects of different aging temperatures on the retention factors (k) and selectivity factors (α) of *n*-alkanes and ester mixtures are shown in Figure 12. The k and α values for the TPP column changed little over the temperature range tested, even when the column was aged at 260°C. This indicated that the TPP column was thermally stable to at least 260 °C. The GC temperatures used to separate the PCB, PAH, PAE, and *n*-alkane mixture also confirmed that the TPP column could be used at up to 260°C. Overall, in terms of service life, the TPP column should be used at <260°C. The results described above indicated that the GC TPP column was thermally stable.

The repeatability of the results acquired using a column will determine the separation precision. This precision was assessed by determining the relative standard deviations of the retention times of *n*-alkanes and esters. As shown in Table 3, the run-to-run and column-to-column relative standard deviations were <0.05% and <4.96%, respectively. These results indicated that the TPP column gave a good degree of precision.

**TABLE 3:** Repeatability of retention times for separation of n-alkane and ester mixtures on TPP column.

	Run to run(*n* = 5)		Column-to-column(*n* = 3)	
	Mean(t_R_)	RSD(%)	Mean(t_R_)	RSD(%)
Methyl heptanoate	3.97	0.02%	3.84	4.96
Decane	4.18	0.05%	4.05	5.63
Methyl caprylate	5.41	0.02%	5.24	3.94
Undecane	5.65	0.03%	5.48	4.35
Methyl nonanoate	6.90	0.02%	6.72	3.35
Dodecane	7.15	0.02%	6.97	3.58
p-Methyl ethylbenzoate	8.26	0.01%	8.11	3.12
Tridecane	8.61	0.03%	8.44	3.28
Methyl undecanate	9.76	0.02%	9.57	2.63
n-tetradecane	10.01	0.02%	9.82	2.74
Methyl Laurate	11.11	0.02%	10.93	2.53
n-Pentadecane	11.37	0.02%	11.18	2.67

## Conclusion

A new GC stationary phase, TPP, gave a high column efficiency, was nonpolar and very thermally stable, and gave good repeatability. The TPP stationary phase had a high resolving ability and gave good peak shapes for various mixtures of nonpolar and polar, aliphatic and aromatic, and acidic to basic compounds. The TPP column was found to be more selective and to give higher resolutions than the HP-5MS column for alicyclic and aromatic molecules because of ring-matching and π–π stacking interactions. PCBs were found to be retained longer by the TPP column than the HP-5MS column because of π–π electron–donor–acceptor interactions between the PCBs and TPP. PCB pairs with similar boiling points were separated by the TPP column (because of differences in PCB planarity) but could not be separated by the HP-5MS column. The results indicated that the TPP column could avoid the disadvantages of the HP-5MS column when separating certain compounds.

## Data Availability

The original contributions presented in the study are included in the article and Supplementary Material, further inquiries can be directed to the corresponding author.
