# Supplementary material for: Capillary Gas Chromatographic Separation Performances of a Tetraphenyl Porphyrin Stationary Phase
Source: Front Chem. 2022 Feb 23;10:800922. doi: 10.3389/fchem.2022.800922 (PMC8905518; doi:10.3389/fchem.2022.800922)
Supplement: Supplementary file 1 [file DataSheet3.docx]

Supplementary Materials

S3 Chromatography to evaluate RSD run to run and column to column


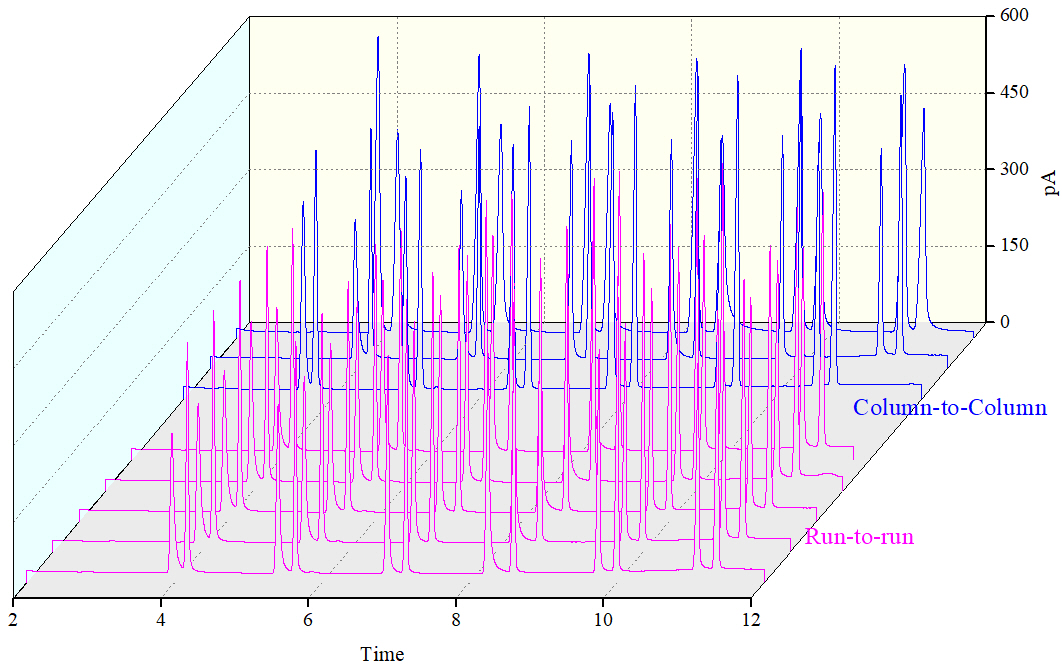


Fig. S3-1 Chromatography to evaluate RSD of run to run and column to column
