# Supplementary material for: Capillary Gas Chromatographic Separation Performances of a Tetraphenyl Porphyrin Stationary Phase
Source: Front Chem. 2022 Feb 23;10:800922. doi: 10.3389/fchem.2022.800922 (PMC8905518; doi:10.3389/fchem.2022.800922)
Supplement: Supplementary file 2 [file DataSheet2.docx]

Supplementary Materials

S2 Compounds tested in the manusript


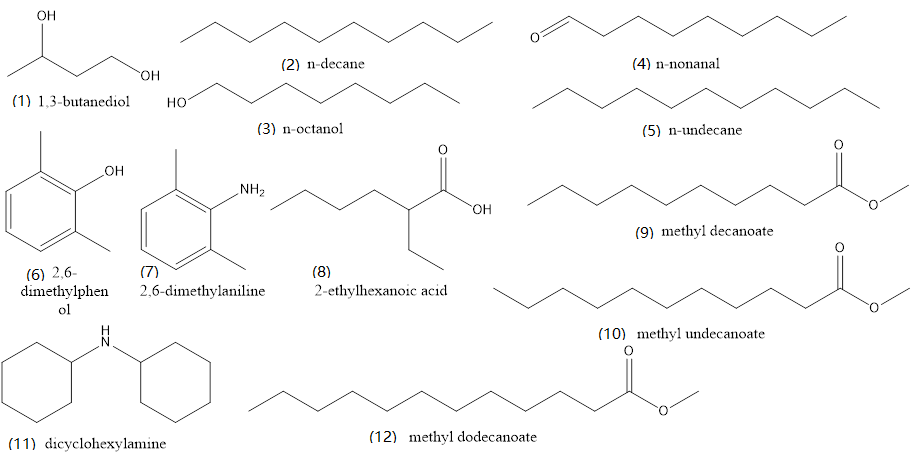


Fig. S2-1 Compounds tested in Fig. 4

(1) 1,3-butanediol, (2) n-decane, (3) n-octanol, (4) n-nonanal, (5) n-undecane, (6) 2,6-dimethylphenol, (7) 2,6-dimethylaniline, (8) 2-ethylhexanoic acid, (9) methyl decanoate, (10) methyl undecanoate, (11) dicyclohexylamine, (12) methyl dodecanoate


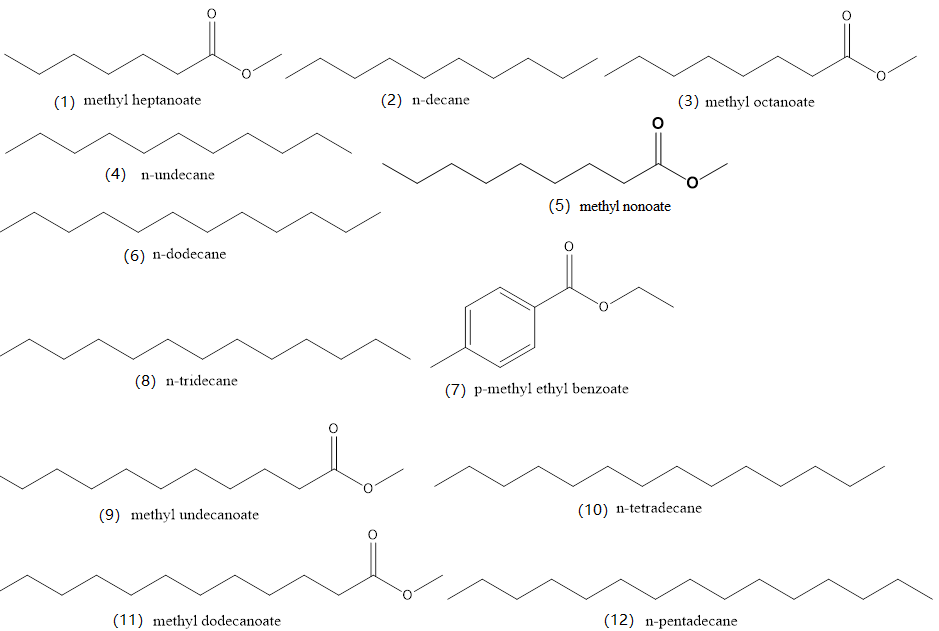


Fig. S2-2 Compounds tested in Fig. 5

(1) methyl heptanoate, (2) n-decane, (3) methyl octanoate, (4) n-undecane, (5) methyl nonoate, (6) n-dodecane, (7) p-methyl ethyl benzoate, (8) n-tridecane, (9) methyl undecanoate, (10) n-tetradecane, (11) methyl dodecanoate, (12) n-pentadecane


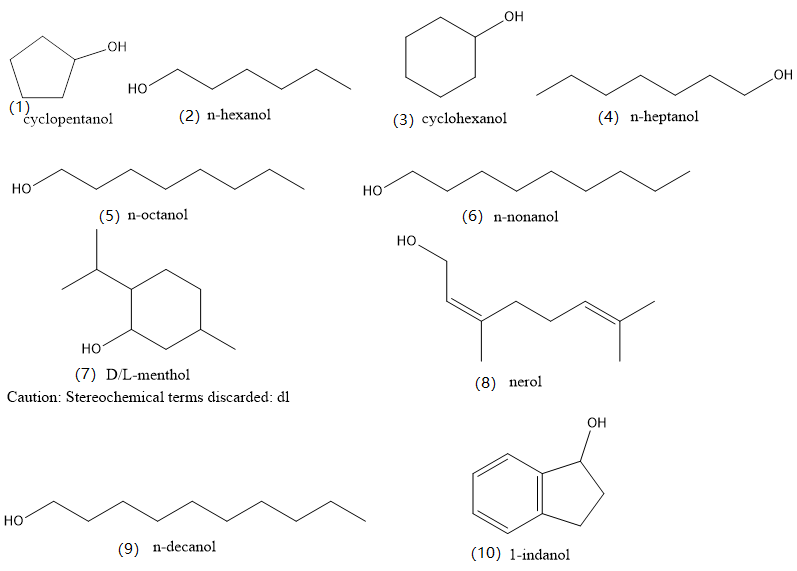


Fig. S2-3 Compounds tested in Fig. 6

(1) cyclopentanol, (2) n-hexanol, (3) cyclohexanol, (4) n-heptanol, (5) n-octanol, (6) n-nonanol, (7) D/L-menthol, (8) nerol, (9) n-decanol, (10) 1-indanol.


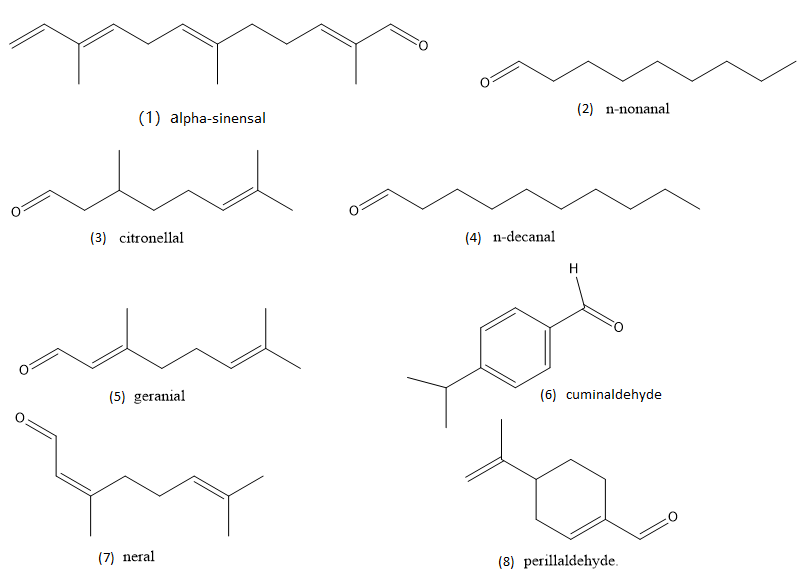


Fig. S2-4 Compounds tested in Fig. 7

sinensal, (2) n-nonanal, (3) citronellal, (4) n-decanal, (5) geranial, (6) cuminaldehyde, (7) neral, (8) perillaldehyde.


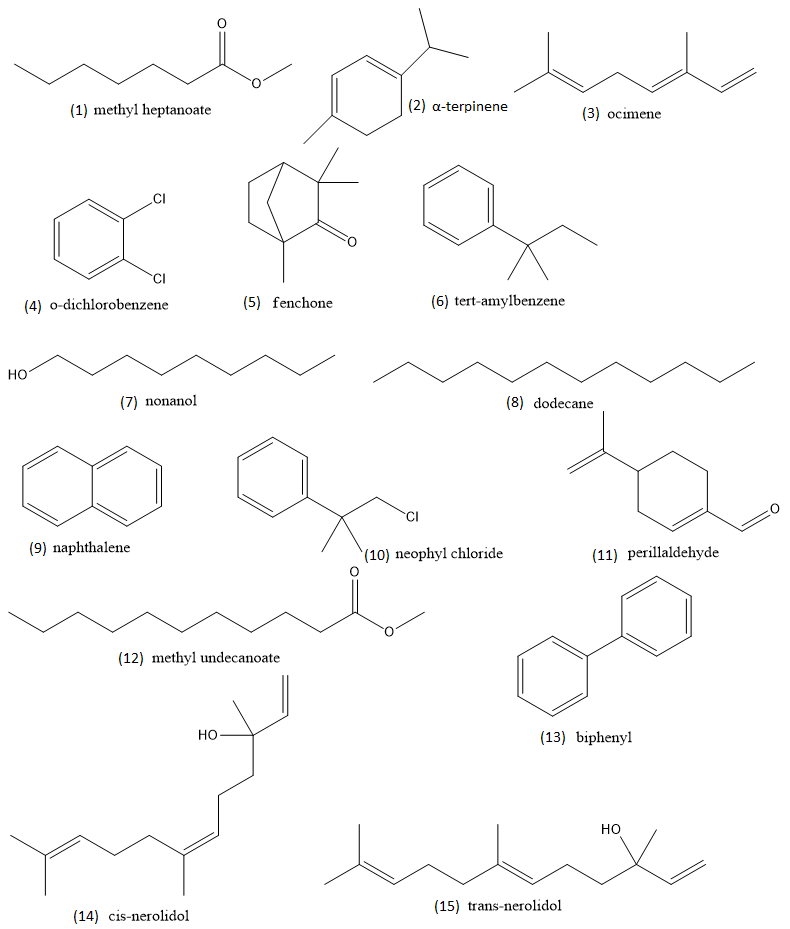


Fig. S2-5 Compounds tested in Fig. 8

(1) methyl heptanoate, (2) α-terpinene, (3) ocimene, (4) o-dichlorobenzene, (5) fenchone, (6) tert-amylbenzene, (7) nonanol, (8) dodecane, (9) naphthalene, (10) neophyl chloride, (11) perillaldehyde, (12) methyl undecanoate, (13) biphenyl, (14) cis-nerolidol, (15) trans-nerolidol.


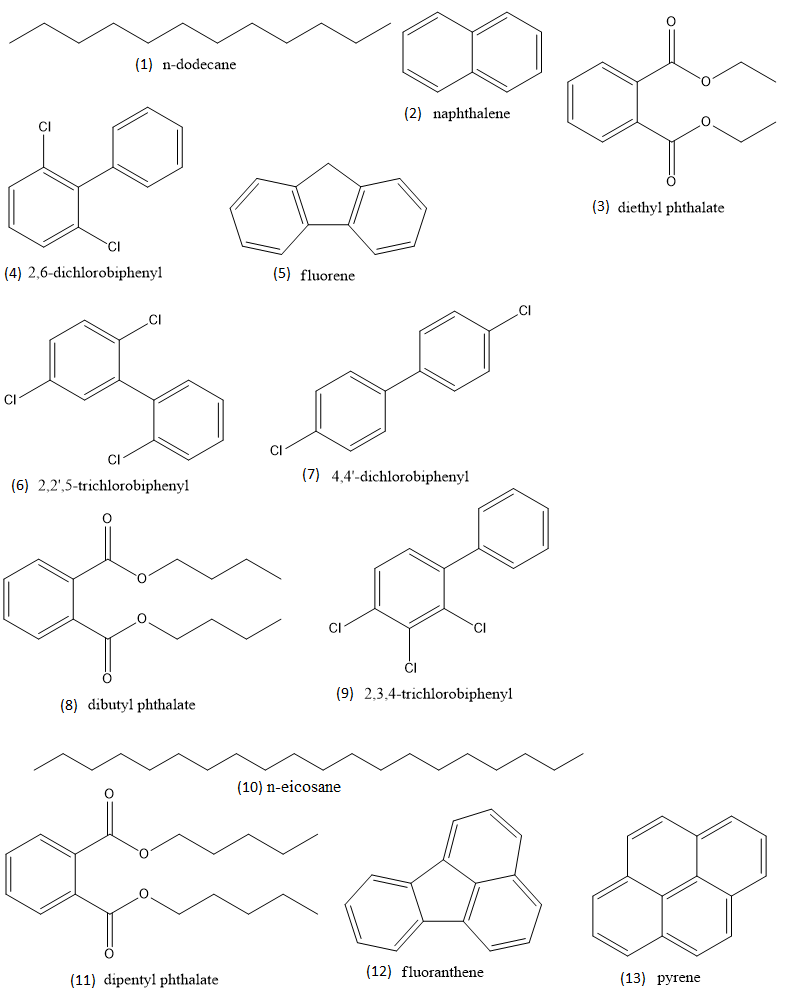


Fig. S2-6 Compounds tested in Fig. 9

(1) n-dodecane, (2) naphthalene, (3) diethyl phthalate, (4) 2,6-dichlorobiphenyl, (5) fluorene, (6) 2,2ʹ,5-trichlorobiphenyl, (7) 4,4ʹ-dichlorobiphenyl, (8) dibutyl phthalate, (9) 2,3,4-trichlorobiphenyl, (10) n-eicosane, (11) dipentyl phthalate, (12) fluoranthene, (13) pyrene.
