# Supplementary material for: Capillary Gas Chromatographic Separation Performances of a Tetraphenyl Porphyrin Stationary Phase
Source: Front Chem. 2022 Feb 23;10:800922. doi: 10.3389/fchem.2022.800922 (PMC8905518; doi:10.3389/fchem.2022.800922)
Supplement: Supplementary file 3 [file DataSheet1.docx]

Supplementary Materials

S1 SEM images of the TPP column

Goal: To prove uniformity of the entire length of the column.

Method: We cut the whole TPP column into multiple fragments. Six segments were selected at random. All six fragments were tested by scan-electron microscopy (SEM), and at least 2 positions were selected to observe for each fragment.

Result and conclusion: Part of the SEM images are listed here. The coating layers is homogeneously distributed across the entire column.


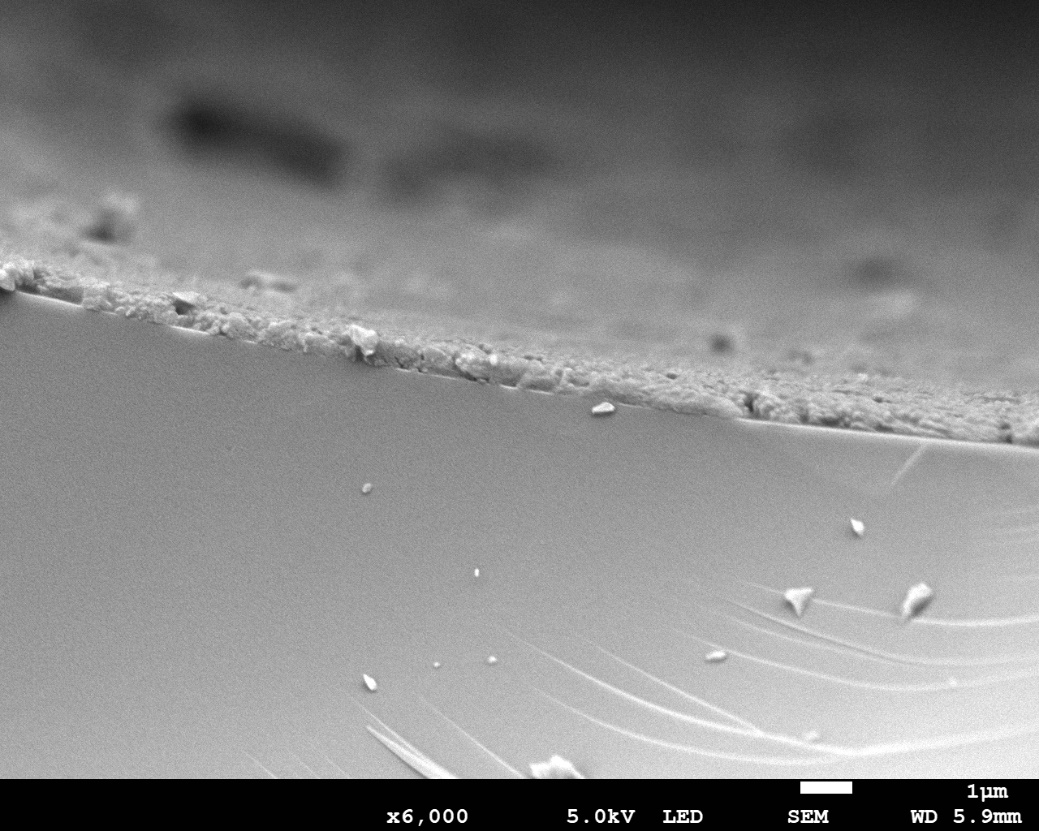


Fig S1-1a SEM image of cross-section of TPP capillary column segment-1


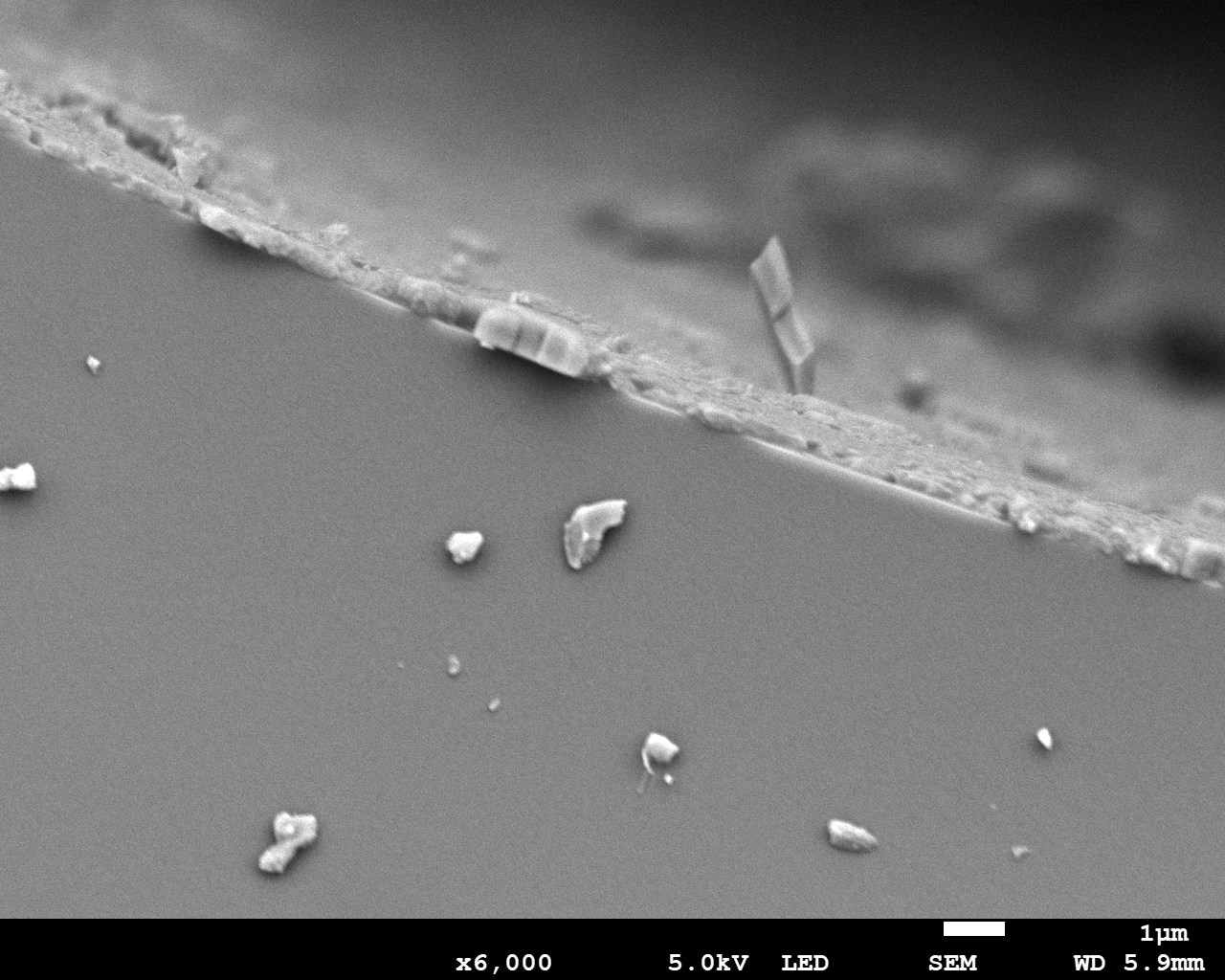


Fig S1-1b SEM image of cross-section of TPP capillary column segment-1


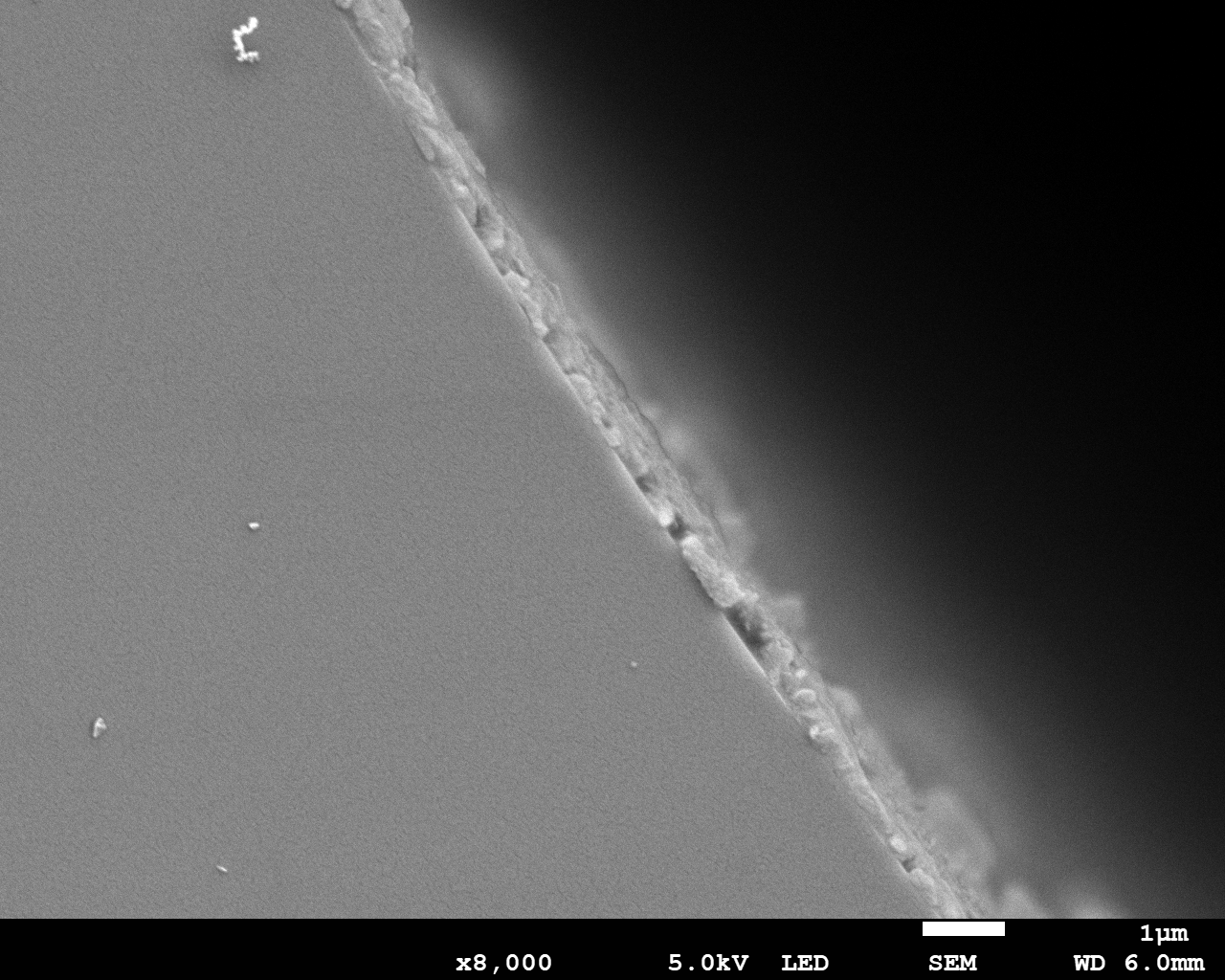


Fig S1-2a SEM image of cross-section of TPP capillary column segment-2


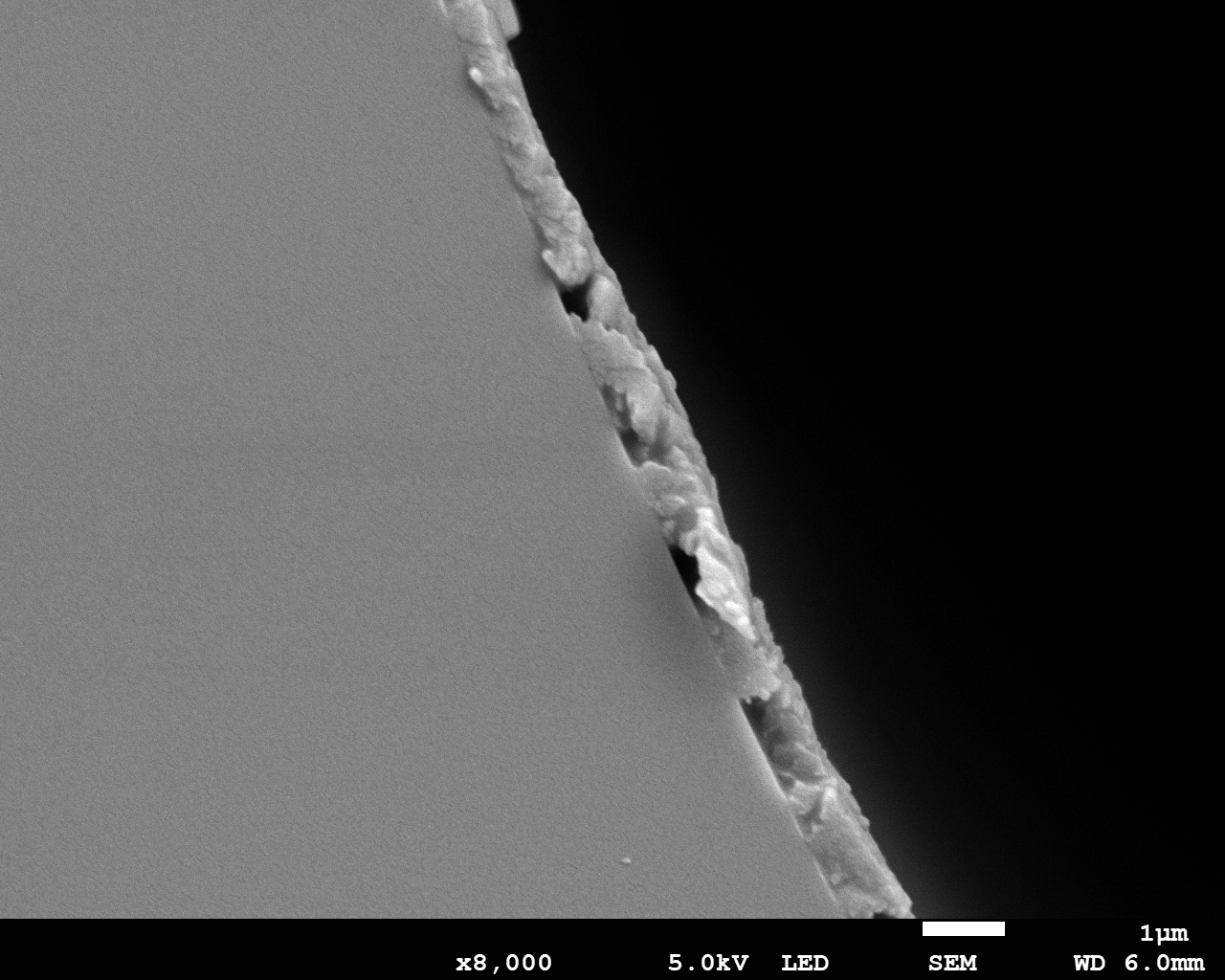


Fig S1-2b SEM image of cross-section of TPP capillary column segment-2


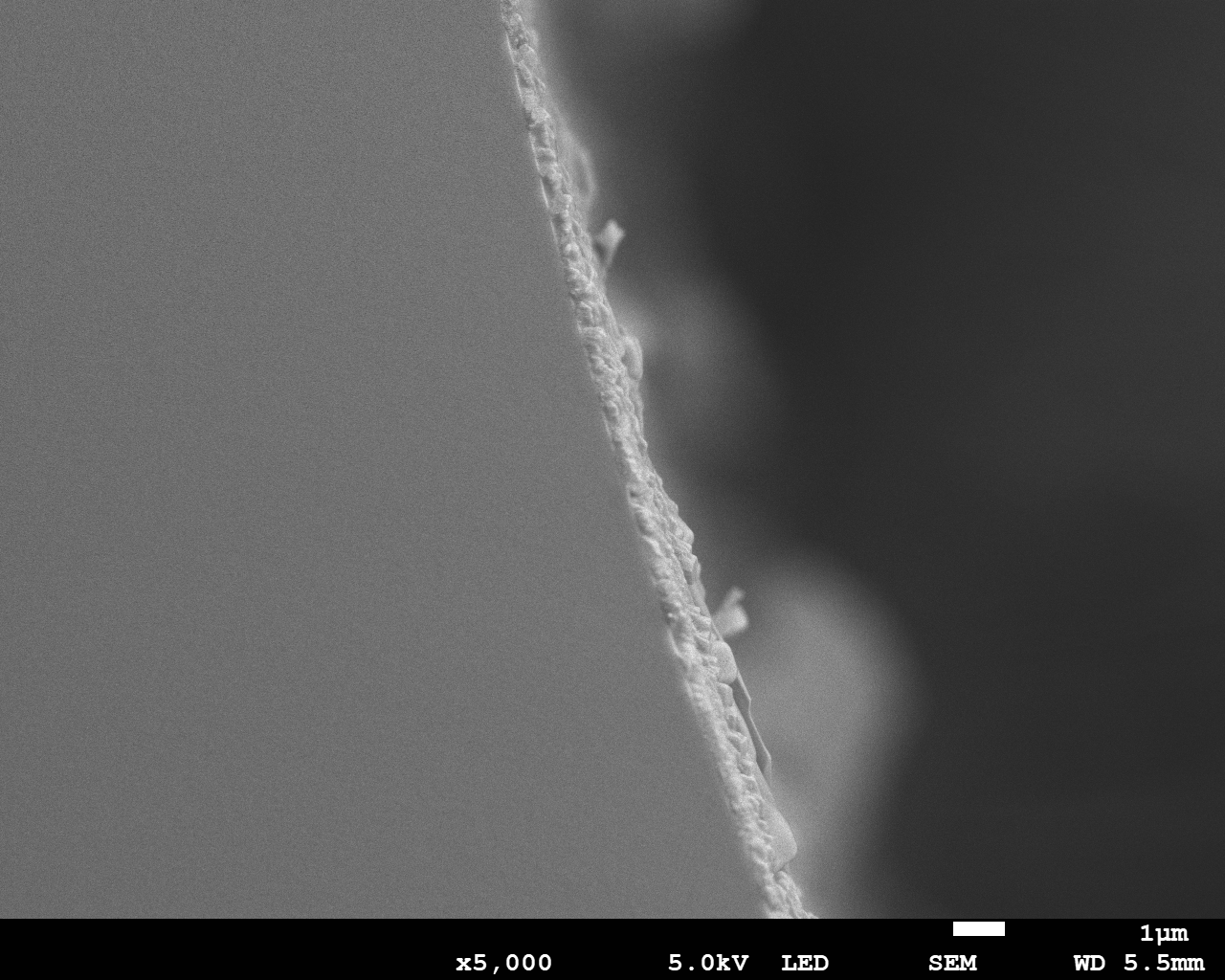


Fig S1-3a SEM image of cross-section of TPP capillary column segment-3


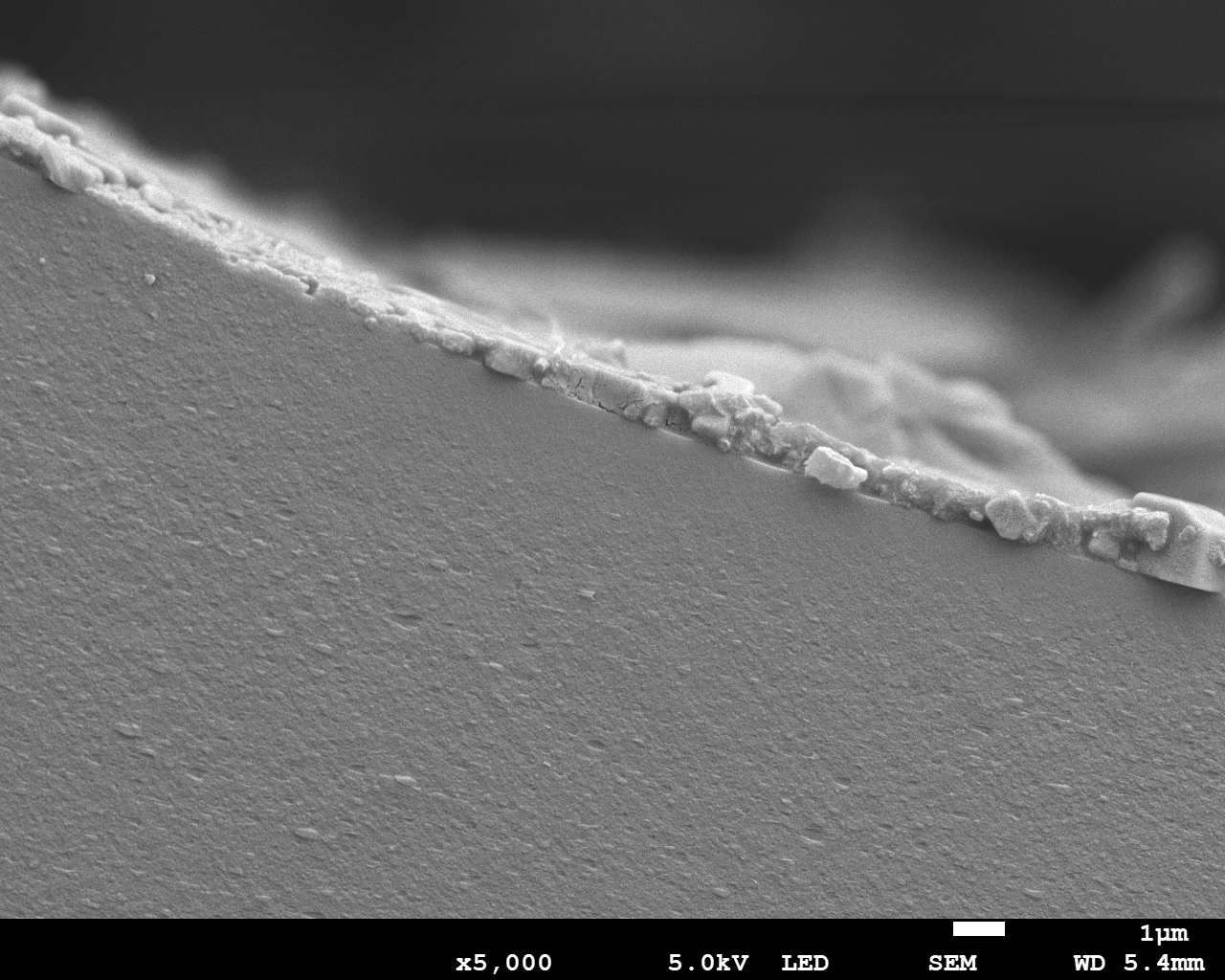


Fig S1-3b SEM image of cross-section of TPP capillary column segment-3


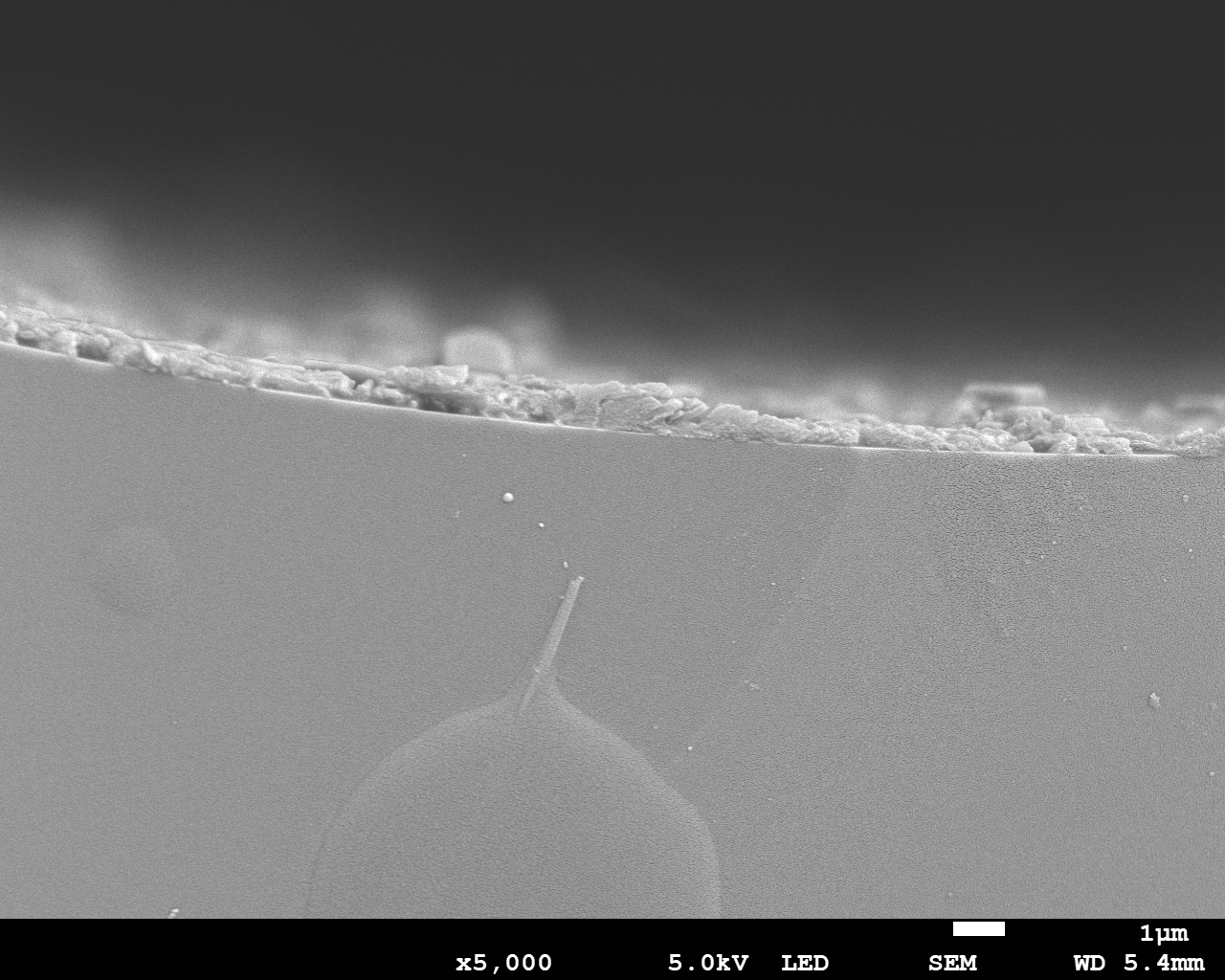


Fig S1-4a SEM image of cross-section of TPP capillary column segment-4


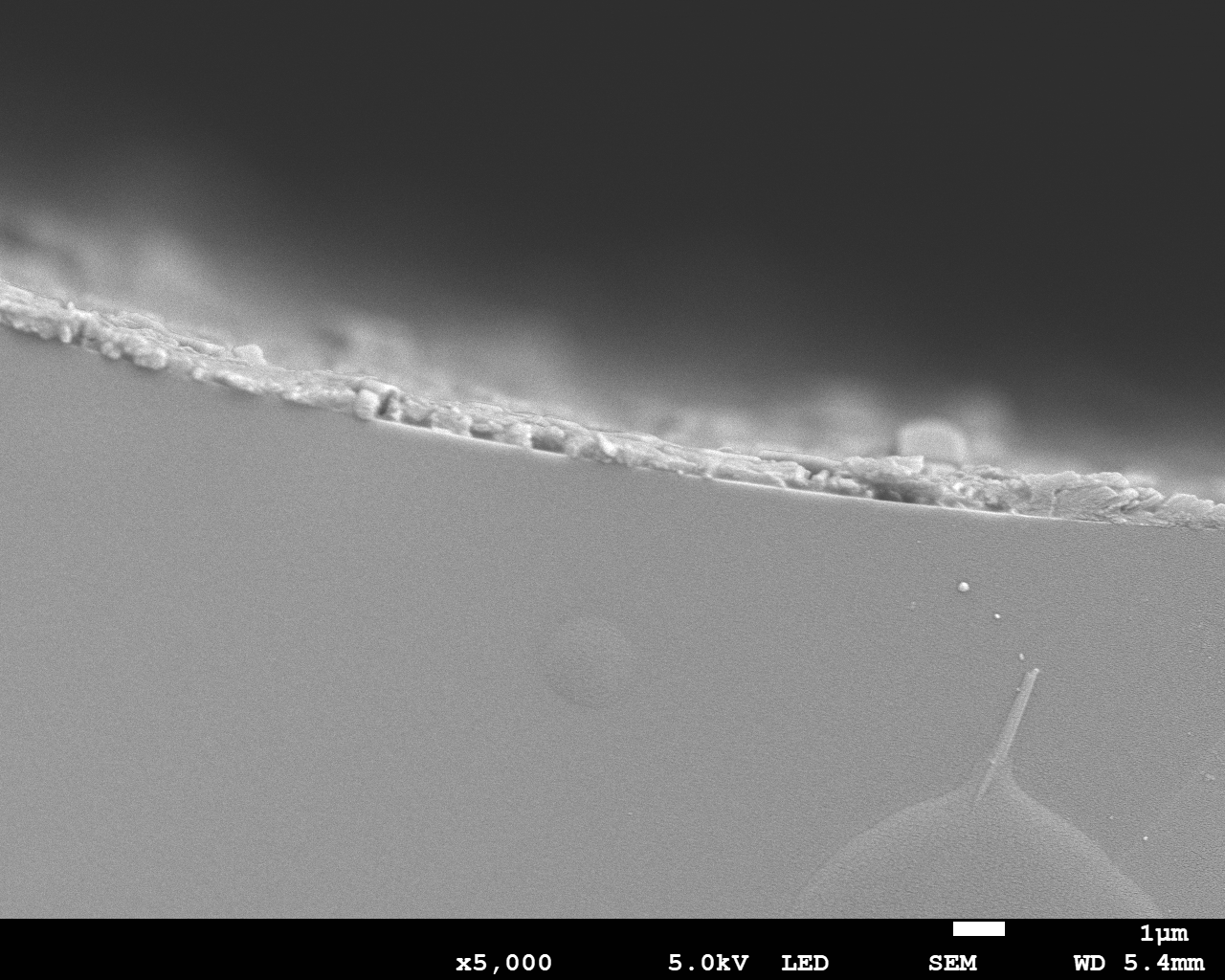


Fig S1-4b SEM image of cross-section of TPP capillary column segment-4
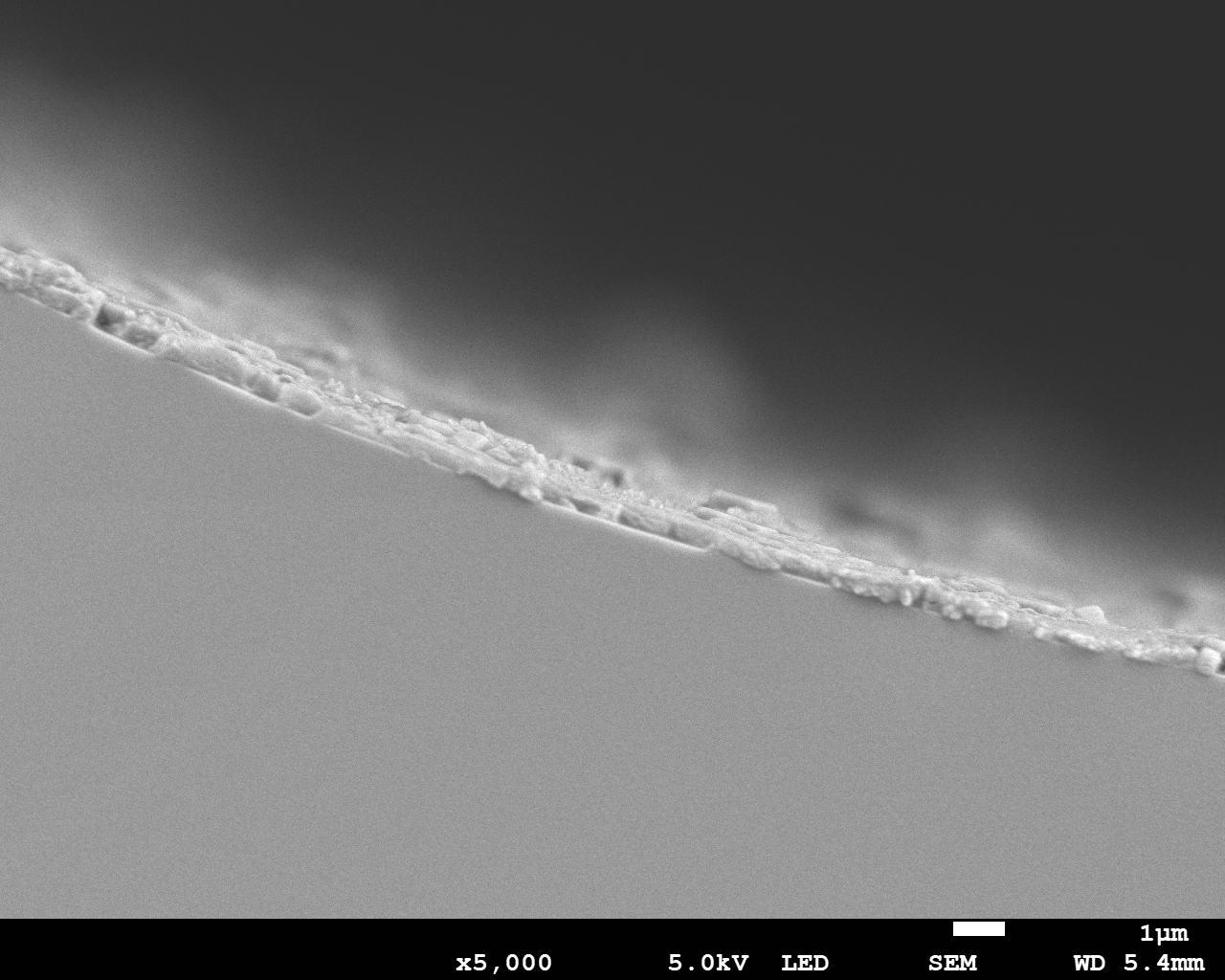


Fig S1-5a SEM image of cross-section of TPP capillary column segment-5


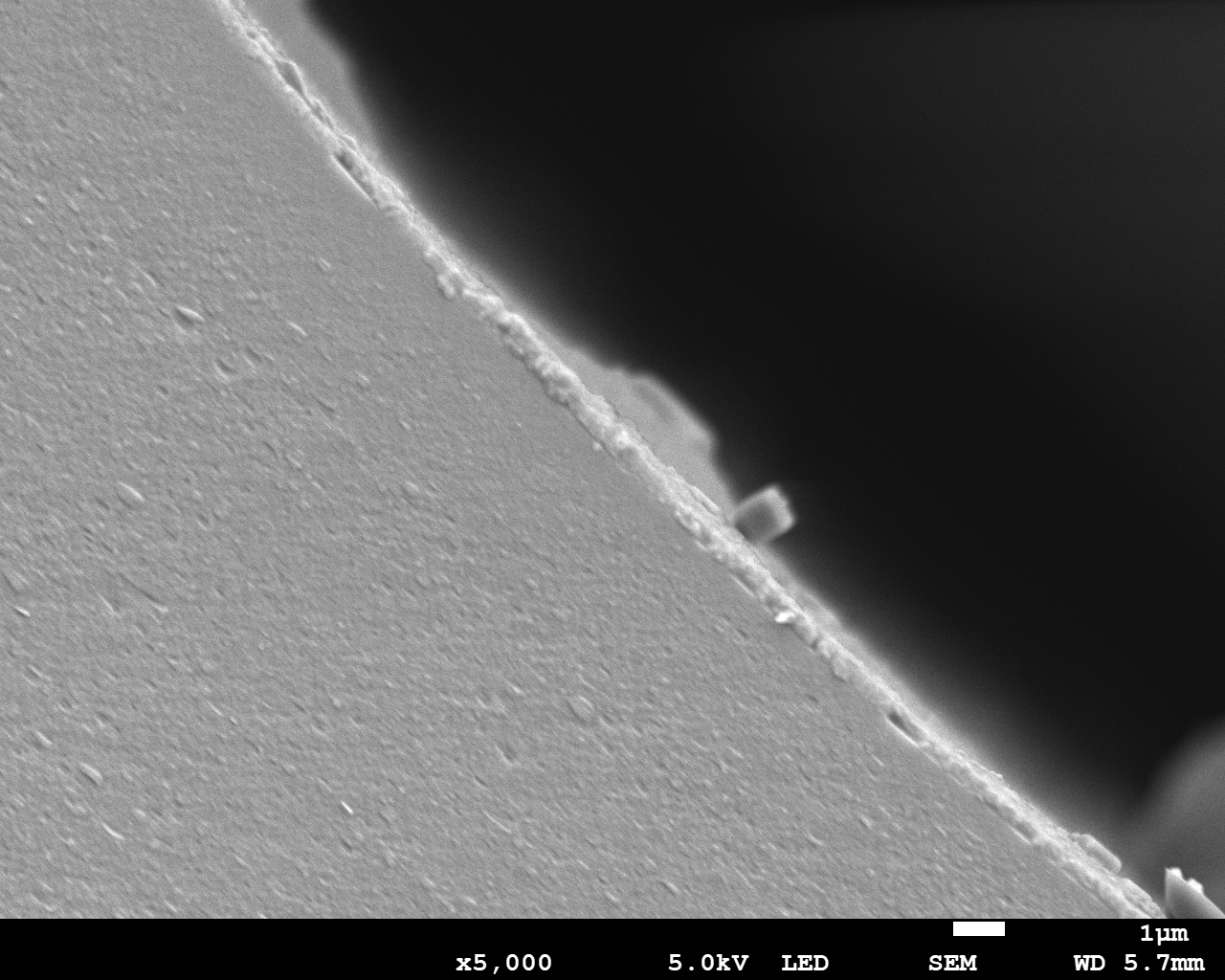


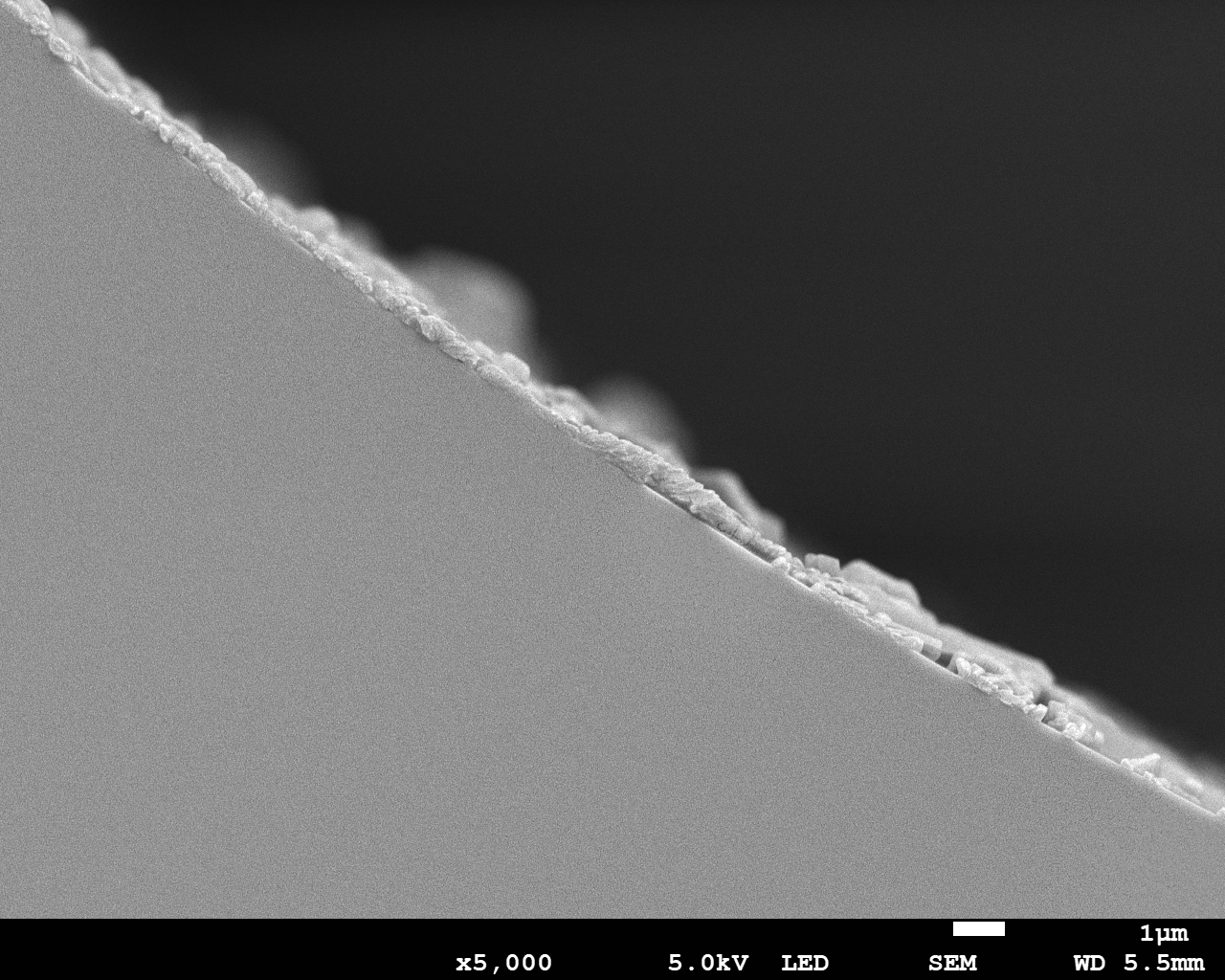


Fig S1-6a SEM image of cross-section of TPP capillary column segment-6


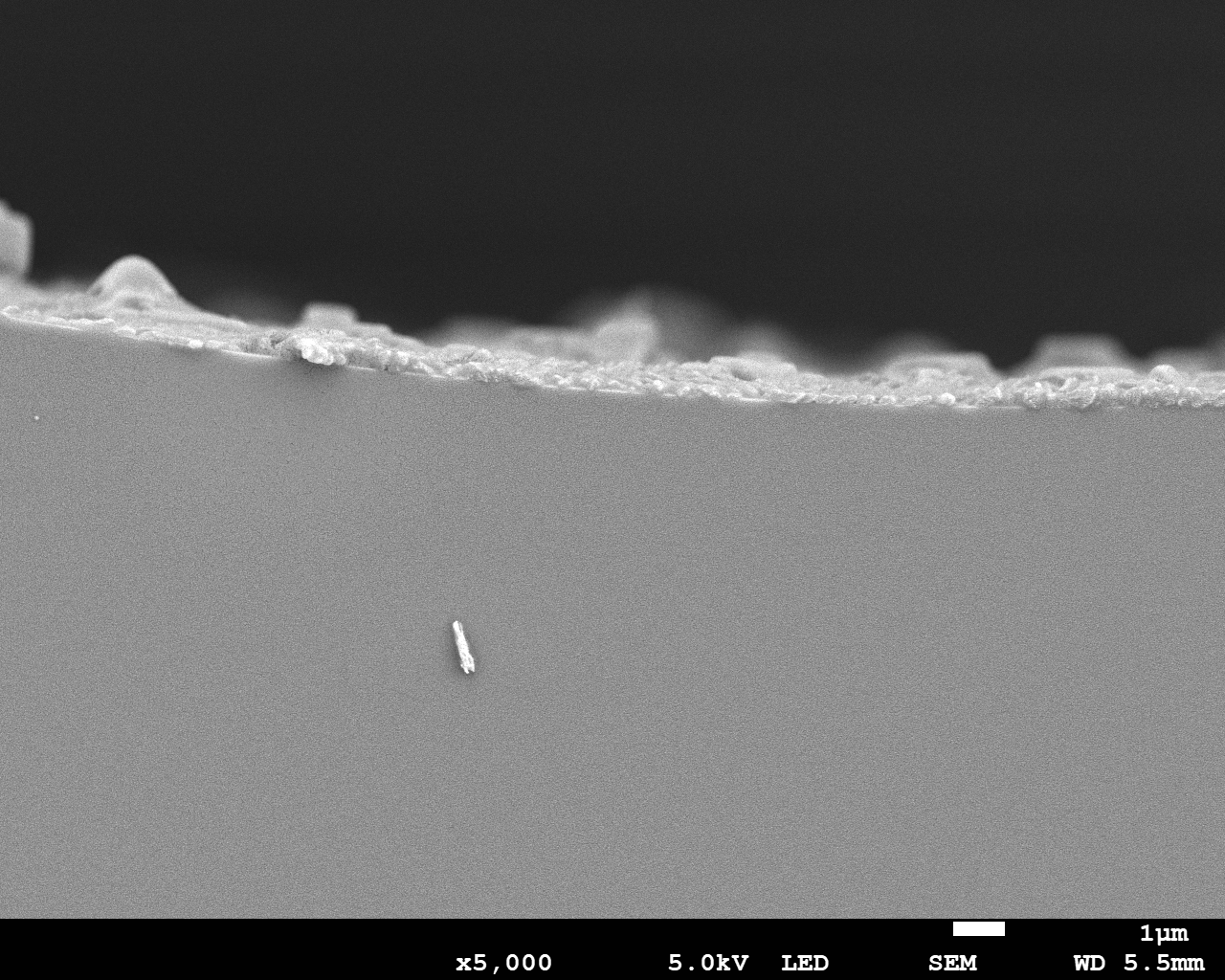


Fig S1-6b SEM image of cross-section of TPP capillary column segment-6
